# The impact of parenthood on environmental attitudes and behaviour: a longitudinal investigation of the legacy hypothesis

**DOI:** 10.1007/s11111-017-0291-1

**Published:** 2017-12-18

**Authors:** Gregory O. Thomas, Rose Fisher, Lorraine Whitmarsh, Taciano L. Milfont, Wouter Poortinga

**Affiliations:** 1 Welsh School of Architecture, Cardiff University, Bute Building, King Edward VII Avenue, Cardiff, Wales CF10 3NB UK; 2 School of Psychology, Cardiff University, 70 Park Place, Cardiff, Wales CF10 3AT UK; 3 Centre for Cross-Cultural Research, School of Psychology, Victoria University of Wellington, Kelburn Parade, Wellington, 6012 New Zealand

**Keywords:** Parenthood, Environmental concern, Environmental attitudes, Environmental behaviour, Longitudinal

## Abstract

Willingness to engage in sustainable actions may be limited by the psychological distance of climate change. In this study, we test the *legacy hypothesis*, which holds that having children leads parents to consider the legacy left to offspring in respect of environmental quality. Using the Understanding Society dataset, a longitudinal survey representative of the UK population (*n* = 18,176), we assess how having children may change people’s individual environmental attitudes and behaviour. Results indicate that having a new child is associated with a small decrease in the frequency of a few environmental behaviours. Only parents with already high environmental concern show a small increase in the desire to act more sustainably after the birth of their first child. Overall, the results do not provide evidence in support of the legacy hypothesis in terms of individual-level environmental attitudes and behaviours. We argue that the transition to parenthood is a time where concern is prioritised on the immediate wellbeing of the child and not on the future environmental threats.

**Electronic supplementary material:**

The online version of this article (10.1007/s11111-017-0291-1) contains supplementary material, which is available to authorized users.

## Introduction

Human behaviour is increasingly causing environmental problems, including biodiversity loss, water pollution and climate change (e.g. IPCC 2014). Addressing these issues requires significant shifts in people’s relationship with the natural world, including making pro-environmental choices. Understanding what influences these choices has been widely studied, and findings highlight a range of individual, social and structural factors that foster and inhibit environmental action (e.g. Stern 2000). One of the hypothesised inhibitors of environmental action is psychological distance, or the belief that environmental problems are too uncertain (hypothetical distance), likely to happen in distant places in the future (geographical and temporal distance), and to people unlike oneself (social distance) (Spence et al. 2012; Evans et al. 2014; Milfont 2010; Schultz et al. 2014). Amongst the dimensions of psychological distance, the temporal aspect seems particularly relevant, given that sustainability definitions explicitly acknowledges temporality, and the main consequences of environmental problems will be borne by future generations. The literature has shown that a focus on the future is associated with greater consideration of environmental protection (Bain et al. 2016; Milfont and Demarque 2015; Milfont et al. 2012a, b). Following this link between future thinking and environmental protection, it has therefore been suggested that having children could be a way of expanding one’s sphere of concern to include future generations. Specifically, having one or more children may lead one to consider the legacy left to offspring in respect of environmental quality, in addition to considering material or financial legacy.

Despite the intuitive appeal of the *legacy hypothesis*, as we refer to it, the hypothesis has received relatively little attention in the literature. The evidence that exists suggests a complex picture, confounded by other factors, and differential impacts on attitudes and behaviours. Furthermore, almost all of the current evidence is cross-sectional. The best way to test the legacy hypothesis is through longitudinal analysis, which enables the examination of changes in environmental attitudes and behaviours following the transition to parenthood. The aim of the current study is to, for the first time, longitudinally investigate the role of having children in people’s individual environmental attitudes and behaviour.

### Parenthood and environmental attitudes and behaviour

The legacy hypothesis, which implies that having children increases concern about the environment and climate change as a result of parents considering the legacy left to offspring in respect of environmental quality, can be linked to a number of theoretical arguments. The *Parental Roles Hypothesi*s (Davidson and Freudenburg 1996, as cited in McCright 2010), and the *Parenthood Status* hypothesis (Blocker and Eckberg 1997), posit that gender differences drive changes in environmental concern as a consequence of parenthood. According to these theoretical positions, mothers become more concerned for the health and safety of their children, and in turn, more concerned for the environment, due to the nurturing role of being a mother. Fathers on the other hand become more concerned with the material well-being of the family and show less concern for the environment. The prediction that women become more concerned for the environment in parenthood has received more support (e.g. Hamilton 1985; Davidson and Freudenburg 1996) than the prediction that men become less concerned for the environment after becoming a father (e.g. Blocker and Eckberg 1989).

Another theoretical argument related to the legacy hypothesis is Erikson’s (1950) generativity concept, or ‘the concern in establishing and guiding the next generation’ (Erikson 1963, p. 267). According to Erikson, generativity is a developmental phase of later life associated with the desire to both leave a social legacy and provide positive guidance to others via intergenerational continuity. Drawing from this perspective, and proposing the idea of *environmental generativity*, Milfont and Sibley (2011) showed that a generativity scale predicted environmental preservation attitudes and ecological behaviour.

Although some studies present findings that are consistent with the legacy hypothesis that parenthood increases environmental concern, many others have not found significant associations of parenthood with environmental concern. For example, McCright (2010) found no associations of parenthood with concern for climate change in either men or women, and Torgler et al. (2008) found no visible parental effect in terms of environmental preferences. Furthermore, Blocker and Eckberg (1997) found that stay-at-home women were less likely to recycle, to be willing to bear the costs of caring for nature and to approve of environmental regulation than women in the workplace. However, it is unclear whether the terms used, ‘homemaker’ and ‘in the labour force’, specifically denote presence or absence of parenthood. Their analyses showed that those who have children are *less* likely to show concern for the environment in a number of measures. Sunblad et al. (2007) also found no effect of having children on risk judgements and worry about climate change, while a more recent study showed that parenthood was associated with higher levels of climate worry but not with perceptions of climate change risk (Ekholm and Olofsson 2016).

Recent qualitative work shows that new mothers’ priorities change to focus on their child’s wellbeing above any other concerns and that environmental choices may or may not be consistent with this. On the one hand, mothers may see energy use as not only necessary for keeping their baby warm and comfortable but also chose greener cleaning products out of concern for their child’s exposure to chemicals (Schäfer et al. 2012; Ha and Williams 2013). At the same time, this and other research (e.g. Thompson et al. 2011) highlights the potential of a transition to parenthood (i.e. the birth of a *first* child) as an important window of opportunity to break and create new habits (Thomas et al. 2016; Verplanken and Roy 2016), although these habits may not naturally be more pro-environmental.

Moving beyond correlational and qualitative analysis, Johnson (2014) investigated parental risk perceptions using experimental methods. When participants were asked to take a parental role in assessing the risk of asbestos, negative views increased. However, when this analysis was repeated, with the additional variable of presence/absence of children at home, it seemed less critical. When the hypothetical judgements were made asking participants to take on the role of a parent, negative reactions significantly increased, but actually having children at home appeared not to have any effect. Another recent experimental study showed that a legacy manipulation—asking participants to write about what they want to be remembered for by future generations—enhanced willingness to engage in environmental behaviours, and increased climate-change beliefs and donations to an environmental organisation (Zaval et al. 2015).

### Complexities in the association between parental status and environmental behaviour

Despite some empirical support for the association between parenthood and environmental behaviour, it is important to note that per capita environmental footprint (e.g. energy use, petrol) increases with family size (e.g. Ha and Williams 2013). These are in addition to other socio-economic aspects of consumption, relating to household income and levels of education (e.g. Poortinga et al. 2004). Although travel patterns are likely to vary between mothers and fathers and with the age of the child (Thompson et al. 2011), car ownership and use tends to increase with the birth of a first child (Prillwitz et al. 2006). While Prillwitz et al. (2006) controlled for household income, employment and education, it is still possible that these effects vary across the socio-economic spectrum. Consumption also increases with family size, leading to greater pressure on natural resources. At the same time, purchase decisions are more likely to be shaped by health and financial concerns than pre-birth. Consequently, some purchases may become more pro-environmental where these choices are also healthier (e.g. organic foods), but equally other choices may be less sustainable if they are cheaper or perceived to be better for their child’s wellbeing (Thompson et al. 2011). On the other hand, self-care behaviours (e.g. showering), seem to change in a more pro-environmental direction with the transition to parenthood, since those with young families often find they have less time for themselves (Thompson et al. 2011). Qualitative research similarly shows that the environmental impacts of the transition to motherhood are complex, with some choices reducing impact and others increasing it (Burningham et al. 2014; Schäfer et al. 2012).

Importantly, the impact of parenthood on environmental behaviour may extend beyond the private sphere (i.e. consumption) to the public sphere (i.e. political and community engagement). For example, Torgler et al. (2008) found that parents are less likely to be a member of a voluntary organisation or volunteer which they infer may be due to time constraints. This so-called time crunch has been widely observed amongst new parents (Thompson et al. 2011), who have to fit existing demands (job, housework etc.) into less available time due to childcare activities and can help explain why time-consuming environmental behaviours (e.g. sorting waste) may decline with the transition to parenthood. More prominent, perhaps, in explaining changes in environmental behaviours is the shift in priorities to consider child wellbeing (and, for some, finances) above any other considerations.

### Aims of the present study

In summary, previous findings are mixed with respect to the relationship between having children on the one hand and engagement in individual-level environmental attitudes and behaviour on the other. Research in this area suggests that there may be gender differences in relation to parenthood, rather than a straightforward relationship between having versus not having children, and that parenthood may have contradictory effects on environmental attitudes and behaviours. Since environmental attitudes are closely linked to environmental behaviours, it is also possible that changes in environmental attitudes could lead to subsequent changes in environmental behaviours after becoming a parent. Furthermore, the vast majority of research has applied cross-sectional research designs. The current study adopts a longitudinal design to allow for analysis of individual-level environmental attitudes and behaviour before versus after having children.

The overall aim of the study is thus to longitudinally investigate the role of having children in people’s environmental attitudes and behaviour. The study has four specific and consecutive objectives. First, it will determine whether *having a child* changes people’s environmental attitudes and behaviour, irrespective of whether the child was a firstborn or not. Second, it will examine whether having a *firstborn* (i.e. becoming a parent for the first time) changes people’s environmental attitudes and behaviour. Third, it will consider whether any changes in attitudes and behaviour are only evident in *new parents with high levels of environmental concern*. Fourth, it will examine whether any changes in attitudes and behaviour are evident in both *new mothers and new fathers*.

## Method

### The understanding society survey

This study makes use of the Understanding Society Survey (USS; University of Essex 2015). The USS is a large-scale panel survey with a core sample of around 40,000 households, representative of the UK population (for more information, see www.understandingsociety.ac.uk). Data are collected in overlapping waves of around 24 months. Wave 1 was collected in 2009–2010, wave 2 in 2010–2011, wave 3 in 2011–2012 and wave 4 in 2012–2013. The USS covers a wide range of topics that are included with varying frequency. A large number of personal and household characteristics, including fertility history as well as major life events such as having children, are included in each wave. Speciality topics, such as environment-related attitudes and behaviour, are measured intermittently on a rotating basis.

This study primarily makes use of wave 1 (conducted from January 2009 to December 2010) and wave 4 (conducted from January 2012 to December 2013). In total, 40,172 individuals took part in wave 1. Of these, 27,193 individuals (67.7%) completed wave 1 through to wave 4. The USS also provides pre-determined weights that allow longitudinal analyses to be representative of the UK population. The final weighted sample size was 18,176. The inclusion of environment-related questions and an explicit recording of the arrival of new children within households allows the legacy hypothesis to be investigated longitudinally with a high-powered and nationallyrepresentative dataset.

### Measures

#### Independent variables

##### Newborn and firstborn children

Each wave of the USS records the arrival of new children in the household by asking whether the individual has had any new children since the last interview. We used these variables in waves 2, 3 and 4, to create a binary variable indicating if a person had reported the birth of a new child during the course of the survey. To determine if a person reported a firstborn child, we first assessed whether a person reported having ever had/mothered any children. We combined the new variable indicating a person had no children at wave 1 with the previously derived variable, indicating that a new child was born during the course of the survey. The variable indicating that a person experienced a firstborn child was then merged with the median split of wave 1 environmental concern (see details below), so that people with firstborn children and high environmental attitudes were selected. Lastly, the indicator variable for having a firstborn child was then merged with gender, to create a variable indicating a respondent who become a new mother during the survey.

Table 1 presents the number of people who reported having a newborn child between waves 1 and 4. A total of 1656 respondents reported a newborn child during the different waves of the survey, approximately 9% of the total sample. Amongst people reporting a new child between waves 2 and 4 of the survey, 740 (4.1%) indicated they had no previous children and were experiencing their firstborn child. Amongst those experiencing a firstborn child, we identified 441 (2.4%) new parents with scores above the median for environmental concern, and 361 (2%) first-time mothers (compared with 379 first-time fathers), to further test the possibility that having a new child would enhance environmental views and/or behaviours.

**Table 1:** Number and proportion of people classified according to their reported arrival of newborn children during the survey (new born), including subgroups for first-time parents (new parent), first-time parents with high environmental concern (new eco-parent) and first-time mothers (new mother)

Classification	Description	Number	% of sample
Newborn	Respondent had a newborn child	1656	9.1
New parent	Respondent had firstborn child	740	4.1
New eco-parent	Respondent had firstborn child and above median environmental attitudes	441	2.4
New mother	Respondent had firstborn child and is female	361	2.0

##### Environmental concern

Wave 1 included nine questions on environmental concern, derived from the 2007 and 2009 Defra surveys of public attitudes to the environment (Defra 2007; Thornton 2009). Responses to the questions formed an adequate scale of environmental concern (Cronbach’s *α* = .67), where higher scores indicate stronger environmental concern. We used a median split to identify two groups of respondents with respective low and high levels of environmental concern. The nine items are listed below:

- The so-called environmental crisis facing humanity has been greatly exaggerated.
- Climate change is beyond control—it is too late to do anything about it.
- The effects of climate change are too far in the future to really worry me.
- I do not believe my behaviour and everyday lifestyle contribute to climate change.
- I would be prepared to pay more for environmentally friendly products.
- If things continue on their current course, we will soon experience a major environmental disaster.
- Any changes I make to help the environment need to fit in with my lifestyle.
- It is not worth me doing things to help the environment if others do not do the same.
- It is not worth Britain trying to combat climate change, because other countries will just cancel out what we do.

##### Socio-demographics

The mean age for the entire sample was 46.21 (SD = 18.15), and results showed that those who reported a newborn child were overall younger (M = 29.19, SD = 6.34) than those who did not (M = 47.92, SD = 18.06), *t*(18,174) = 41.92, *p* < .001, *d* = 1.38. The gender split for the whole sample was 52.6% female, and a chi-squared test showed that the gender proportion for those reporting a newborn child (9.1% for both male and female respondents) was not statistically different, *X* ^2^ (1, 18,176) = 0.04, *p* = .85. The reported income of the sample the month prior to taking part in the survey was £3389.57 (SD = 2701.25), and results showed that those who reported a newborn child had a higher monthly income (M = £3716.04, SD = 2570.46) than those without newborns (M = £3356.84, SD = 2711.92), *t*(18174) = 5.16, *p* < .001, *d* = 0.14.

#### Dependent variables

##### Environmental lifestyle attitudes

Waves 1 and 4 included three items measuring different aspects of environmental lifestyle, each of which was measured in a different way. First, respondents were asked ‘Which of these best describes how you feel about your current lifestyle and the environment?’. This item could be answered using the following options: ‘I’m happy with what I do at the moment’ (lowest value), ‘I’d like to do a bit more to help the environment’ and ‘I’d like to do a lot more to help the environment’ (highest value). Second, respondents were asked ‘Which of these would you say best describes your current lifestyle?’. Here, respondents could answer using a 5-point scale ranging from the lowest score indicating ‘I don’t really do anything that is environmentally-friendly’ to the highest score indicating ‘I’m environmentally-friendly in everything I do’. Third, respondents were asked ‘Do you agree or disagree that being green is an alternative lifestyle, it’s not for the majority?’. Respondents could answer on a 4-point agree-disagree scale where the lowest value indicate agreement that being green is not for the majority, and the highest value indicate disagreement that being green is for the majority. The items were analysed separately because they could not be combined into an internally consistent scale (Cronbach’s *α* for waves 1 and 4 = .25 and .24, respectively).

##### Environmental behaviours

Waves 1 and 4 included the self-reported frequency of engaging in 11 headline environmental behaviours (Defra 2008). Respondents indicated the frequency they engaged in each behaviour using a 5-point scale ranging from ‘always’ (5) to ‘never’ (1). The items were analysed separately because they could not be combined into an internally consistent scale (Cronbach’s *α* for waves 1 and 4 = .55 and .52, respectively). The 11 environmental behaviours are listed below:

- Leave your TV on standby for the night.
- Switch off lights in rooms that are not being used.
- Keep the tap running while you brush your teeth.
- Put more clothes on when you feel cold rather than putting the heating on or turning it up.
- Decide not to buy something because you feel it has too much packaging.
- Buy recycled paper products such as toilet paper or tissues.
- Take your own shopping bag when shopping.
- Use public transport (e.g. bus, train) rather than travel by car.
- Walk or cycle for short journeys less than 2 or 3 miles.
- Car share with others who need to make a similar journey.
- Take fewer flights when possible.

### Statistical analysis

Four sets of linear regression models were constructed to address the objectives of the study. We calculated the change in environmental lifestyle attitudes and change in environmental behaviours between waves 1 and 4 for use as dependent variables.^1^ Changes in both environmental attitudes and behaviours were calculated so that higher scores indicate an increase in holding more positive views on a sustainable lifestyle or an increase in acting sustainably. This means that the dependent variables had a wider range of outcomes than their one-time measurements. That is, the dependent variables of change in behaviour had a 9-point scale ranging from − 4 to + 4 and the dependent variables of change in lifestyle views had a 5-point (ranging from − 2 to + 2), 7-point (ranging from − 3 to + 3) or 9-point (ranging from − 4 to + 4) scale, depending on whether their one-time measurements had 3, 4 and 5 response categories, respectively.

Noting the observed differences in age and income between those with newborns and those without, we included socio-demographics of age and income as covariates in the first step of the model, and also the wave 1 score of the dependent variable to control for regression to the mean effects.

The independent variables were four dummy variables indicating: (1) if respondents had a newborn child during the time of the survey (*newborn status*), (2) if they had a firstborn child (*new parent status*), (3) if respondents had a firstborn child and had above-average environmental concern (*new eco-parent status*) and (4) if they had a firstborn child and were female (*new mother status*). With three measures of environmental attitudes and 11 environmental behaviours, this created four series of regressions that predicted changes in each of the 14 dependent variables. Caution is required when interpreting results from a large number of analyses, as increasing the number of independent analyses also increases the probability of encountering a type I error, where we may mistakenly reject the null hypothesis (Tabachnick and Fidell 2013, p. 53). To control for inflated error rates from multiple comparisons, we apply the Holm-Bonferroni correction (Holm 1979) to all significant *p* values. The Holm-Bonferroni method offers greater statistical power by sequentially applying a Bonferroni correction to each significance value in ascending order of size, while relaxing the Bonferroni correction as the number of comparisons decrease. We first apply the strictest Bonferroni correction to the smallest observed significance value, and then for the second smallest value this correction is relaxed, and so forth.

#### Data availability

Data are available through the UK Data Archive under the end-user licence (10.5255/UKDA-SN-6614-6).

## Results

### Newborn status

Regression analyses were run predicting changes in environmental attitudes and changes in environmental behaviour between waves 1 and 4 of the survey. We first included covariates of age, monthly income and the baseline measures of each dependent variable in the model, but to conserve space these are not reported. The full results of the regression analyses can be found in Supporting information 1. We then included a dummy variable indicating whether the respondents had/mothered a child during the survey (*n* = 1656), with results summarised in Table 2. Analysis indicated that having a newborn child was a significant predictor of a *reduction* in several measures of environmental attitudes and behaviours from waves 1 to 4, and not an increase as expected. Applying the Holm-Bonferroni correction to all *p* values < .05 nullified the significance of several coefficients. However, three predictions remained significant after controlling for inflated error rates, with results indicating that the status of having a newborn child led to a very small decrease (all *β*s = − 0.03) in the frequency of three environmental behaviours (‘wear more clothes instead of more heating’, ‘use public transport instead of car’ and ‘carshare with others’), compared with those who did not report a newborn child.

**Table 2:** Regression coefficients of newborn status for changes in environmental attitudes and sustainable behaviours

Coefficient of newborn status for	*B*	Se	Beta	Sig.	Sig. (corr)
Desire to increase greener lifestyle	0.03	0.01	0.01	.079	
Strength of green lifestyle	− 0.06	0.02	− 0.02	.007	.350
Green lifestyle as ‘alternative’	− 0.04	0.02	− 0.01	.029	.999
Leave TV on standby	− 0.08	0.05	− 0.01	.074	
Switch off unused lights	− 0.06	0.02	− 0.01	.017	.799
Turn off tap when brushing teeth	− 0.03	0.04	− 0.01	.475	
Wear more clothes instead of more heating	*− 0.13*	*0.03*	*− 0.03*	*< .001*	*.005*
Not purchase products with too much packaging	0.03	0.02	0.01	.209	
Buy recycled paper products	0.04	0.03	0.01	.192	
Take own shopping bags	− 0.04	0.03	− 0.01	.264	
Use public transport instead of car	*− 0.11*	*0.03*	*− 0.03*	*< .001*	*.012*
Walk/cycle short journeys	0.04	0.03	0.01	.302	
Carshare with others	*− 0.15*	*0.04*	*− 0.03*	*< .001*	*.003*
Take fewer flights	0.00	0.04	0.00	.921	

Note: Covariates of age, annual income, and baseline view/behaviour not shown to conserve space. Original significance values given, with Holm-Bonferroni correction applied to significant results. Values set in italics indicate significant effects that remained after controlling for inflated error rates

### New parent status

Regression analyses were then run using a dummy variable indicating whether respondents reported a newborn child and also had not previously had/mothered any children (*n* = 740). Again covariates of age, annual income and baseline measures of each dependent variable were included, but not reported here to conserve space (see Supporting information 1 for the full regression output). Results for new parent status on environmental attitudes and behaviours are shown in Table 3.

**Table 3:** Regression coefficients of new parent status for changes in environmental attitudes and environmental behaviours

Coefficient of new parent status for	*B*	Se	Beta	Sig.	Sig. (corr)
Desire to increase greener lifestyle	0.02	0.02	0.01	.234	
Strength of green lifestyle	− 0.07	0.03	− 0.02	.020	.920
Green lifestyle as ‘alternative’	− 0.02	0.03	− 0.01	.450	
Leave TV on standby	− 0.04	0.06	− 0.01	.491	
Switch off unused lights	*− 0.12*	*0.03*	*− 0.02*	*< .001*	*.016*
Turn off tap when brushing teeth	− 0.08	0.05	− 0.01	.167	
Wear more clothes instead of more heating	− 0.13	0.05	− 0.02	.003	.153
Not purchase products with too much packaging	0.04	0.03	0.01	.297	
Buy recycled paper products	0.02	0.05	0.00	.637	
Take own shopping bags	− 0.02	0.05	0.00	.734	
Use public transport instead of car	− 0.11	0.04	− 0.02	.007	.357
Walk/cycle short journeys	0.05	0.05	0.01	.346	
Carshare with others	− 0.01	0.05	0.00	.843	
Take fewer flights	0.02	0.06	0.00	.680	

Note: Covariates of age, annual income, and baseline view/behaviour not shown to conserve space. Original significance values given, with Holm-Bonferroni correction applied to significant results. Values set in italics indicate significant effects that remained after controlling for inflated error rates

Results indicate that the status of becoming a new parent was again linked to *reductions* in several environmental attitudes and behaviours. Application of Holm-Bonferroni correction identified only one significant relationship after controlling for inflated error rates. The status of becoming a first-time parent was a significant, but very small (*β* = − 0.02), predictor of reduced frequency of switching off lights in rooms that were not being used, compared with those who did not become first-time parents.

### New eco-parent status

We then specified the regression model to include the dummy variable indicating that respondents had become a first-time parent and also had above-average environmental concern at wave 1 of the survey (*n* = 441). Covariates of age, annual income and baseline measures of each dependent variable were included (see Supporting information 1 for full regression output), and results for new eco-parent status on changes on environmental attitudes and behaviours are summarised in Table 4.

**Table 4:** Regression coefficients of new eco-parent status for changes in environmental attitudes and environmental behaviours

Coefficient of new eco-parent status for	*B*	Se	Beta	Sig.	Sig. (corr)
Desire to increase greener lifestyle	*0.09*	*0.03*	*0.02*	*< .001*	*.017*
Strength of green lifestyle	− 0.03	0.04	− 0.01	.457	
Green lifestyle as ‘alternative’	0.08	0.03	0.02	.020	.900
Leave TV on standby	− 0.07	0.08	− 0.01	.385	
Switch off unused lights	− 0.06	0.04	− 0.01	.166	
Turn off tap when brushing teeth	− 0.03	0.07	0.00	.635	
Wear more clothes instead of more heating	0.00	0.06	0.00	.949	
Not purchase products with too much packaging	0.07	0.04	0.01	.133	
Buy recycled paper products	0.13	0.06	0.01	.034	
Take own shopping bags	− 0.05	0.06	− 0.01	.430	
Use public transport instead of car	− 0.08	0.05	− 0.01	.162	
Walk/Cycle short journeys	0.10	0.06	0.01	.091	
Carshare with others	− 0.08	0.06	− 0.01	.241	
Take fewer flights	0.14	0.07	0.02	.052	

Note: Covariates of age, annual income and baseline view/behaviour not shown to conserve space. Original significance values given, with Holm-Bonferroni correction applied to significant results. Values set in italics indicate significant effects that remained after controlling for inflated error rates

Results show that becoming a new parent with high environmental concern predicted a *reduction* in frequency of one environmental behaviour and an increase in two environmental attitudes. However, after the application of Holm-Bonferroni corrections to control for inflated error rates, the decrease in behaviour (buying recycled paper products) and increase in one environmental attitude (perception of a green lifestyle as suitable for the majority) were no longer significant. One increase in environmental attitudes remained significant, indicating that new parents with high environmental concern show a very small increase (*β* = 0.02) in their desire to increase their environmental lifestyle.

### New mother status

Finally, we ran the regression models to include a dummy variable indicating whether respondents had become a first-time parent and was female (i.e. becoming a new mother, *n* = 361). Covariates of age, annual income and baseline measures of each dependent variable were included, and a summary of the results for new mother status predicting changes in environmental attitudes and behaviour are presented in Table 5.

**Table 5:** Regression coefficients of new mother status for changes in environmental attitudes and environmental behaviours

Coefficient of new mother status for	*B*	Se	Beta	Sig.	Sig. (corr)
Desire to increase greener lifestyle	− 0.01	0.03	0.00	.630	
Strength of green lifestyle	− 0.08	0.04	− 0.01	.083	
Green lifestyle as ‘alternative’	0.02	0.04	0.00	.633	
Leave TV on standby	− 0.01	0.09	0.00	.903	
Switch off unused lights	− 0.12	0.05	− 0.02	.012	.576
Turn off tap when brushing teeth	− 0.13	0.08	− 0.01	.082	
Wear more clothes instead of more heating	− 0.05	0.06	− 0.01	.443	
Not purchase products with too much packaging	0.05	0.05	0.01	.319	
Buy recycled paper products	0.01	0.07	0.00	.900	
Take own shopping bags	− 0.11	0.07	− 0.01	.119	
Use public transport instead of car	0.01	0.06	0.00	.840	
Walk/cycle short journeys	0.04	0.07	0.00	.555	
Carshare with others	− 0.01	0.07	0.00	.902	
Take fewer flights	− 0.05	0.08	− 0.01	.549	

Note: Covariates of age, annual income and baseline view/behaviour not shown to conserve space. Original significance values given, with Holm-Bonferroni correction applied to significant results.

Results indicate that becoming a new mother was predictive of a very small *decrease* in the frequency of one environmental behaviour. Again, however, the application of the Holm-Bonferroni correction for inflated error rates rendered this relationship non-significant.

## Discussion

This paper explored whether having children leads to changes in individual-level environmental attitudes and behaviours, possibly as an effect of having greater consideration for future generations (the ‘legacy hypothesis’). Using the Understanding Society Survey, changes in three environmental attitude items and the frequency of 11 environmental behaviours were assessed for those who had children in between two waves of data collection. We examined four groups of people: those who had at least one new child (irrespective of whether this was a firstborn or not), those who became a parent for the first time, first-time parents with high environmental concern and first-time mothers. Our analysis showed only small changes in individual-level environmental attitudes and behaviours following people having a new child. In contrast with expectations from the legacy hypothesis, all changes were negative, indicating the environmental behaviours were performed less often. The only observed positive change was an increase in the desire to act more sustainably amongst first-time parents who already had a high level of environmental concern. Overall, the results do not provide support for the legacy hypothesis. Where there are any changes, these are more likely to be negative, suggesting that having a child *reduces* self-reported environmental behaviours.

Several studies have demonstrated that perceiving the impact of climate change as distant, whether in terms of physical distance, timescale, social distance or uncertainty in its effects, reduces people’s willingness to engage with sustainable actions, and that encouraging people to consider the future induces an increase in sustainable actions. The legacy hypothesis suggests that psychological distance is reduced in relation to environmental problems and inducing sustainable actions, where the birth of a child makes a new parent consider their child’s future. Yet, there is no strong empirical evidence for the hypothesis, at least not for individual-level environmental attitudes and behaviours. Our results indicate that only amongst eco-minded new parents there may a very small increase in desire to act more sustainably, but this is not reflected in their behaviours. Importantly, the analysis was conducted using a large-scale and high-quality, longitudinal dataset, broadly supporting that parenthood does not enhance sustainable views and actions.

Although there is an intuitive appeal to the legacy hypothesis, it is worth noting that the transition to becoming a parent raises several barriers to acting sustainably, which may explain some of the findings. Becoming a new parent can change a person’s entire outlook, as the new child becomes the central focus in a person’s life (Burningham et al. 2014). Qualitative work highlights that new mothers’ primary concerns are focused on the immediate health and wellbeing of their children, and these considerations often outweigh concerns for sustainability (Ha and Williams 2013). Other reports also find that new parents highlight the comfort and health of their babies as their primary concern, which then involves using more energy (e.g. using more heating and more frequent use of washing appliances) and driving more for comfort and safety (Schäfer et al. 2012). While new parents may increase their purchase of organic foods and shopped for local produces, these behaviours are more likely to be motivated by health (i.e. avoiding chemicals) rather than environmental reasons (Ha and Williams 2013; Schäfer et al. 2012). In addition, the time available to new parents is constrained, as they navigate personal responsibilities and caring for their new child (Thompson et al. 2011), which then can reduce the motivation or ability to act sustainably. Our results show that parenthood is linked to a reduced frequency of some environmental behaviours. These behaviours are consistent with a greater need for caring for a child than for sustainability, such as turning up household heating, increased driving and avoiding public transport or car sharing, and keeping lights turned on (possibly as night lights).

The transition to parenthood involves changing priorities and actions, often where behaviours are focused on producing benefits for the child’s immediate well-being, and not future planning for environmental protection. The consideration of future generations can encourage greater concern for climate change (Milfont and Demarque 2015; Zaval et al. 2015), but considering one aspect of parenthood may not capture the full impact of becoming a parent. Indeed, some experimental work has found that imagining oneself as a parent can generate protective and environmental views, but the actual status of being a parent was not a significant predictor (Johnson 2014). Considering children and future generations may bridge a psychological distance between a person and the environment, but the reality and pressures of raising a new child may outweigh the motivation for sustainability.

The implications of this research suggest that the transition to parenthood is not a defining characteristic in promoting greater sustainability in terms of individual-level environmental attitudes and behaviour. People may not naturally develop greater concern for the environment after having a new child. We find that even those with strong views may only develop a marginal increase in their *desire* to act more sustainably after having a child, and then this intention may not be reflective of actual behaviour change. We note that our results do not dismiss the idea of environmental legacy as an effective way of promoting sustainability after a child is born. Having a child is a major life event, and several psychology scholars have argued that such periods of change may be ideal for interventions to develop new habits (Verplanken and Roy 2016; Thomas et al. 2016). Future research should explore opportunities for the promotion of sustainability during the parenthood transition in more detail. People with high environmental concern did show an increased desire to live a more sustainable lifestyle after becoming a new parent (albeit this was a very small effect) and may welcome support for positive changes during this time (Schäfer et al. 2012). Moreover, it is possible that the transition to parenthood might not impact environmentally relevant actions directly but indirectly, as illustrated by a study showing that parental status moderated the link between support for climate-change actions and voting intentions, with support for climate-change actions predicting *increased* support for a centre-left political party and *decreased* support for a centre-right party but only for people with children (Milfont et al. 2012a, b).

Although our analysis used a high-quality longitudinal, nationally representative dataset, it is not without limitations. We used self-reported data on a limited number of individual-level environmental attitudes and behaviours, which may be open to recall or self-presentation biases, but may also not accurately capture the effect of parenthood. There is an extremely wide range of environmental behaviours a person may undertake, and our analysis was limited to 11 individual-level behaviours, although they are considered headline behaviours for a number of different areas (Defra 2008). Having children leads to a change in priorities, and parents may prioritise sustainability for their new children in a host of different ways (Ha and Williams 2013; Burningham et al. 2014). Instead of the individual-level behaviours measured here, parents may instead have chosen to enact environmental behaviours directly linked to their children. In particular, the use of nappies is the most prominent discussion in parenthood and sustainability, with parents facing the choice of reusable or disposable nappies (Thompson et al. 2011).

Perhaps equally important, environmentalism is not expressed in individual consumer behaviours only. According to the social movement literature, social change is driven by collective action, where individuals and resources are mobilised to garner support for political struggles. Stern et al. (1999) and Stern (2000) present a model of the basis of support for environmental movements, in which they distinguish between private-sphere environmentalism, and activist and non-activist behaviours in the public sphere. The current paper specifically focused on individual private-sphere consumer behaviours. It is possible that the effect of parenthood may be found in more collectively expressed participation in social movements and environmental citizenship.

Furthermore, the study specifically focused on how having children may affect how people think about the environment as a result of considering the legacy left to their offspring. According to Erikson (1950, 1963), generativity, or the concern in establishing and guiding the next generation, develops in middle adulthood and involves considering the best interests of children. Importantly, having children is not a prerequisite nor a guarantee for generativity. In terms of our research, the average age of the parents of newborns was too low for Erikson’s 7th stage of development in which to expect generativity. It is possible that considerations of generativity will not have developed at such an early stage of having children. Likewise, the implication of generativity not being limited to those who have children, is that environmental legacy may develop more generally in the second half of life. Observations that age and environmental concern are inversely related would however argue against such effects (Honnold 1984; Marquart-Pyatt 2008). More research is needed to clearly disentangle legacy from generativity effects in the environmental domain.

Overall, there appears to be a very limited amount of research into the implications of parenthood on individual-level environmental behaviours, intentions and beliefs, and we would welcome future work on this topic, in particular with regard to a broader range of environmental as well as generativity effects across the lifespan.

## Electronic supplementary material

ESM 1 (DOCX 170 kb)

## Footnotes

1 Ordinary least squares (OLS) linear regression is appropriate when the dependent variable is normally distributed (Tabachnick and Fidell 2013, p.466), and changes in the lifestyle views and sustainable behaviours met this assumption. We compared OLS and Ordinal Regression approaches with items that may not fulfil normal distribution assumptions. Results between the two approaches were highly similar, and therefore only report the results of the linear regression here.
