# Supplementary material for: The impact of parenthood on environmental attitudes and behaviour: a longitudinal investigation of the legacy hypothesis
Source: Popul Environ. 2017 Dec 18;39(3):261–76. doi: 10.1007/s11111-017-0291-1 (PMC5846977; doi:10.1007/s11111-017-0291-1)
Supplement: Supplementary file 1 — (DOCX 170 kb) [file 11111_2017_291_MOESM1_ESM.docx]

# Supporting Information 1: Full regression output

The Understanding Society dataset uses codes for the different variables, which are listed below. The change in environmental lifestyle attitudes and behaviours between Wave 1 and 4 were used as dependent variables in the regression analyses. Two analyses were conducted for each dependent variable (Model 1 and 2). Model 1 included the covariates of age, income, and Wave 1 score of the dependent variable (e.g. “Wave 1 ftst”) to control for regression to the mean effects. Model 2 expands Model 1 by adding a dummy variable for either newborn status, new parent status, new eco-parent status or new mother status. The regressions are listed according to the analyses for newborn status, new parent status, new eco-parent status and new mother status, respectively. B = unstandardised regression coefficient; SE = standard error; β = standardised regression coefficient; CI= confidence interval; R^2^ = explained variance in the dependent variable.

| **Code** | **Description** |
| --- | --- |
| *Environmental lifestyle attitudes* | |
| ftst | Which of these best describes how you feel about your current lifestyle and the environment? |
| crlf | Which of these would you say best describes your current lifestyle? |
| grn | Do you agree or disagree that being green is an alternative lifestyle, it’s not for the majority? |
| *Environmental behaviours* | |
| envhabit1 | Leave your TV on standby for the night |
| envhabit2 | Switch off lights in rooms that aren’t being used |
| envhabit3 | Keep the tap running while you brush your teeth |
| envhabit4 | Put more clothes on when you feel cold rather than putting the heating on or turning it up |
| envhabit5 | Decide not to buy something because you feel it has too much packaging |
| envhabit6 | Buy recycled paper products such as toilet paper or tissues |
| envhabit7 | Take your own shopping bag when shopping |
| envhabit8 | Use public transport (e.g., bus, train) rather than travel by car |
| envhabit9 | Walk or cycle for short journeys less than 2 or 3 miles |
| envhabit10 | Car share with others who need to make a similar journey |
| envhabit11 | Take fewer flights when possible |

## Newborn Status

| **Model** | **Dependent Variable: Change in ftst** | **B** | **SE** | **β** | **t** | **p** | **Lower 95%CI** | **Upper 95%CI** | **R^2^** |
| --- | --- | --- | --- | --- | --- | --- | --- | --- | --- |
| 1 | (Constant) | 1.019 | 0.017 |  | 60.706 | 0.000000 | 0.986 | 1.052 | .332 |
|  | Age | -0.003 | 0.000 | -0.083 | -13.184 | 0.000000 | -0.003 | -0.002 |  |
|  | Income | 0.000 | 0.000 | 0.012 | 2.003 | 0.045163 | 0.000 | 0.000 |  |
|  | Wave 1 Ftst | -0.650 | 0.007 | -0.584 | -94.55 | 0.000000 | -0.664 | -0.637 |  |
| 2 | (Constant) | 1.011 | 0.017 |  | 58.09 | 0.000000 | 0.977 | 1.045 | .332 |
|  | Age | -0.003 | 0.000 | -0.080 | -12.094 | 0.000000 | -0.003 | -0.002 |  |
|  | Income | 0.000 | 0.000 | 0.013 | 2.045 | 0.040834 | 0.000 | 0.000 |  |
|  | Wave 1 Ftst | -0.650 | 0.007 | -0.584 | -94.548 | 0.000000 | -0.664 | -0.637 |  |
|  | Newborn | 0.025 | 0.014 | 0.011 | 1.759 | 0.078557 | -0.003 | 0.053 |  |
| **Model** | **Dependent Variable: Change in crlf** | **B** | **SE** | **β** | **t** | **p** | **Lower 95%CI** | **Upper 95%CI** | **R^2^** |
| 1 | (Constant) | 1.454 | 0.025 |  | 58.844 | 0.000000 | 1.406 | 1.503 | .302 |
|  | Age | 0.005 | 0.000 | 0.103 | 15.723 | 0.000000 | 0.005 | 0.006 |  |
|  | Income | 0.000 | 0.000 | 0.002 | 0.369 | 0.711800 | 0.000 | 0.000 |  |
|  | Wave 1 crlf | -0.623 | 0.007 | -0.562 | -88.074 | 0.000000 | -0.637 | -0.609 |  |
| 2 | (Constant) | 1.473 | 0.026 |  | 57.346 | 0.000000 | 1.423 | 1.523 | .302 |
|  | Age | 0.005 | 0.000 | 0.097 | 14.296 | 0.000000 | 0.004 | 0.006 |  |
|  | Income | 0.000 | 0.000 | 0.002 | 0.305 | 0.760544 | 0.000 | 0.000 |  |
|  | Wave 1 crlf | -0.623 | 0.007 | -0.563 | -88.1 | 0.000000 | -0.637 | -0.610 |  |
|  | Newborn | -0.058 | 0.022 | -0.017 | -2.667 | 0.007649 | -0.100 | -0.015 |  |

| **Model** | **Dependent Variable: Change in grn** | **B** | **SE** | **β** | **t** | **p** | **Lower 95%CI** | **Upper 95%CI** | **R^2^** |
| --- | --- | --- | --- | --- | --- | --- | --- | --- | --- |
| 1 | (Constant) | 1.671 | 0.023 |  | 71.308 | 0.000000 | 1.625 | 1.717 | .334 |
|  | Age | -0.002 | 0.000 | -0.044 | -6.995 | 0.000000 | -0.003 | -0.001 |  |
|  | Income | 0.000 | 0.000 | 0.039 | 6.2 | 0.000000 | 0.000 | 0.000 |  |
|  | Wave 1 grn | -0.638 | 0.007 | -0.586 | -93.864 | 0.000000 | -0.652 | -0.625 |  |
| 2 | (Constant) | 1.684 | 0.024 |  | 69.627 | 0.000000 | 1.637 | 1.732 | .335 |
|  | Age | -0.002 | 0.000 | -0.048 | -7.323 | 0.000000 | -0.003 | -0.002 |  |
|  | Income | 0.000 | 0.000 | 0.039 | 6.154 | 0.000000 | 0.000 | 0.000 |  |
|  | Wave 1 grn | -0.639 | 0.007 | -0.586 | -93.895 | 0.000000 | -0.652 | -0.625 |  |
|  | Newborn | -0.039 | 0.018 | -0.014 | -2.164 | 0.030461 | -0.074 | -0.004 |  |

| **Model** | **Dependent Variable: Change in envhabit1** | **B** | **SE** | **β** | **t** | **p** | **Lower 95%CI** | **Upper 95%CI** | **R^2^** |
| --- | --- | --- | --- | --- | --- | --- | --- | --- | --- |
| 1 | (Constant) | 1.276 | 0.048 |  | 26.806 | 0.000000 | 1.183 | 1.370 | .236 |
|  | Age | 0.008 | 0.001 | 0.077 | 11.373 | 0.000000 | 0.007 | 0.009 |  |
|  | Income | 0.000 | 0.000 | -0.026 | -3.856 | 0.000116 | 0.000 | 0.000 |  |
|  | Wave 1 Envhabit1 | -0.539 | 0.007 | -0.488 | -73.904 | 0.000000 | -0.553 | -0.524 |  |
| 2 | (Constant) | 1.304 | 0.050 |  | 26.051 | 0.000000 | 1.206 | 1.402 | .236 |
|  | Age | 0.008 | 0.001 | 0.073 | 10.337 | 0.000000 | 0.006 | 0.009 |  |
|  | Income | 0.000 | 0.000 | -0.026 | -3.906 | 0.000094 | 0.000 | 0.000 |  |
|  | Wave 1 Envhabit1 | -0.539 | 0.007 | -0.489 | -73.926 | 0.000000 | -0.553 | -0.525 |  |
|  | Newborn | -0.079 | 0.045 | -0.012 | -1.786 | 0.074181 | -0.167 | 0.008 |  |

| **Model** | **Dependent Variable: Change in envhabit2** | **B** | **SE** | **β** | **t** | **p** | **Lower 95%CI** | **Upper 95%CI** | **R^2^** |
| --- | --- | --- | --- | --- | --- | --- | --- | --- | --- |
| 1 | (Constant) | -1.237 | 0.025 |  | -50.012 | 0.000000 | -1.285 | -1.188 | .398 |
|  | Age | 0.003 | 0.000 | 0.053 | 9.001 | 0.000000 | 0.003 | 0.004 |  |
|  | Income | 0.000 | 0.000 | -0.034 | -5.758 | 0.000000 | 0.000 | 0.000 |  |
|  | Wave 1 Envhabit2 | 0.730 | 0.007 | 0.638 | 109.283 | 0.000000 | 0.717 | 0.743 |  |
| 2 | (Constant) | -1.218 | 0.026 |  | -46.912 | 0.000000 | -1.269 | -1.167 | .398 |
|  | Age | 0.003 | 0.000 | 0.049 | 7.885 | 0.000000 | 0.002 | 0.004 |  |
|  | Income | 0.000 | 0.000 | -0.034 | -5.814 | 0.000000 | 0.000 | 0.000 |  |
|  | Wave 1 Envhabit2 | 0.729 | 0.007 | 0.637 | 109.188 | 0.000000 | 0.716 | 0.743 |  |
|  | Newborn | -0.056 | 0.023 | -0.014 | -2.394 | 0.016671 | -0.102 | -0.010 |  |

| **Model** | **Dependent Variable: Change in envhabit3** | **B** | **SE** | **β** | **t** | **p** | **Lower 95%CI** | **Upper 95%CI** | **R^2^** |
| --- | --- | --- | --- | --- | --- | --- | --- | --- | --- |
| 1 | (Constant) | 1.335 | 0.039 |  | 34.027 | 0.000000 | 1.258 | 1.411 | .240 |
|  | Age | 0.005 | 0.001 | 0.052 | 7.851 | 0.000000 | 0.004 | 0.006 |  |
|  | Income | 0.000 | 0.000 | 0.023 | 3.449 | 0.000564 | 0.000 | 0.000 |  |
|  | Wave 1 Envhabit3 | -0.459 | 0.006 | -0.489 | -75.102 | 0.000000 | -0.471 | -0.447 |  |
| 2 | (Constant) | 1.344 | 0.041 |  | 32.602 | 0.000000 | 1.263 | 1.424 | .240 |
|  | Age | 0.005 | 0.001 | 0.051 | 7.293 | 0.000000 | 0.003 | 0.006 |  |
|  | Income | 0.000 | 0.000 | 0.023 | 3.43 | 0.000604 | 0.000 | 0.000 |  |
|  | Wave 1 Envhabit3 | -0.459 | 0.006 | -0.489 | -75.101 | 0.000000 | -0.471 | -0.447 |  |
|  | Newborn | -0.027 | 0.038 | -0.005 | -0.714 | 0.475474 | -0.102 | 0.048 |  |

| **Model** | **Dependent Variable: Change in envhabit4** | **B** | **SE** | **β** | **t** | **p** | **Lower 95%CI** | **Upper 95%CI** | **R^2^** |
| --- | --- | --- | --- | --- | --- | --- | --- | --- | --- |
| 1 | (Constant) | -1.549 | 0.034 |  | -45.55 | 0.000000 | -1.616 | -1.483 | .334 |
|  | Age | -0.001 | 0.001 | -0.014 | -2.226 | 0.026021 | -0.002 | 0.000 |  |
|  | Income | 0.000 | 0.000 | -0.003 | -0.553 | 0.580469 | 0.000 | 0.000 |  |
|  | Wave 1 Envhabit4 | 0.644 | 0.007 | 0.577 | 94.847 | 0.000000 | 0.631 | 0.657 |  |
| 2 | (Constant) | -1.510 | 0.035 |  | -42.572 | 0.000000 | -1.579 | -1.440 | .334 |
|  | Age | -0.002 | 0.001 | -0.021 | -3.291 | 0.001000 | -0.003 | -0.001 |  |
|  | Income | 0.000 | 0.000 | -0.004 | -0.652 | 0.514370 | 0.000 | 0.000 |  |
|  | Wave 1 Envhabit4 | 0.644 | 0.007 | 0.578 | 94.943 | 0.000000 | 0.631 | 0.658 |  |
|  | Newborn | -0.127 | 0.032 | -0.025 | -3.935 | 0.000084 | -0.191 | -0.064 |  |

| **Model** | **Dependent Variable: Change in envhabit2** | **B** | **SE** | **β** | **t** | **p** | **Lower 95%CI** | **Upper 95%CI** | **R^2^** |
| --- | --- | --- | --- | --- | --- | --- | --- | --- | --- |
| 1 | (Constant) | -2.766 | 0.037 |  | -75.333 | 0.000000 | -2.838 | -2.694 | .341 |
|  | Age | 0.000 | 0.000 | 0.000 | 0.002 | 0.998725 | -0.001 | 0.001 |  |
|  | Income | 0.000 | 0.000 | 0.015 | 2.476 | 0.013287 | 0.000 | 0.000 |  |
|  | Wave 1 Envhabit5 | 0.625 | 0.007 | 0.584 | 95.184 | 0.000000 | 0.612 | 0.638 |  |
| 2 | (Constant) | -2.776 | 0.038 |  | -73.914 | 0.000000 | -2.850 | -2.703 | .341 |
|  | Age | 0.000 | 0.000 | 0.002 | 0.374 | 0.708776 | -0.001 | 0.001 |  |
|  | Income | 0.000 | 0.000 | 0.016 | 2.507 | 0.012169 | 0.000 | 0.000 |  |
|  | Wave 1 Envhabit5 | 0.625 | 0.007 | 0.584 | 95.19 | 0.000000 | 0.612 | 0.638 |  |
|  | Newborn | 0.030 | 0.024 | 0.008 | 1.255 | 0.209329 | -0.017 | 0.077 |  |

| **Model** | **Dependent Variable: Change in envhabit6** | **B** | **SE** | **β** | **t** | **p** | **Lower 95%CI** | **Upper 95%CI** | **R^2^** |
| --- | --- | --- | --- | --- | --- | --- | --- | --- | --- |
| 1 | (Constant) | -2.420 | 0.041 |  | -59.735 | 0.000000 | -2.499 | -2.340 | .329 |
|  | Age | 0.002 | 0.001 | 0.019 | 2.909 | 0.003626 | 0.001 | 0.003 |  |
|  | Income | 0.000 | 0.000 | 0.016 | 2.503 | 0.012309 | 0.000 | 0.000 |  |
|  | Wave 1 Envhabit6 | 0.628 | 0.007 | 0.575 | 91.058 | 0.000000 | 0.615 | 0.642 |  |
| 2 | (Constant) | -2.435 | 0.042 |  | -57.743 | 0.000000 | -2.518 | -2.352 | .329 |
|  | Age | 0.002 | 0.001 | 0.021 | 3.17 | 0.001530 | 0.001 | 0.003 |  |
|  | Income | 0.000 | 0.000 | 0.016 | 2.532 | 0.011338 | 0.000 | 0.000 |  |
|  | Wave 1 Envhabit6 | 0.628 | 0.007 | 0.575 | 91.069 | 0.000000 | 0.615 | 0.642 |  |
|  | Newborn | 0.044 | 0.033 | 0.009 | 1.305 | 0.191803 | -0.022 | 0.109 |  |

| **Model** | **Dependent Variable: Change in envhabit7** | **B** | **SE** | **β** | **t** | **p** | **Lower 95%CI** | **Upper 95%CI** | **R^2^** |
| --- | --- | --- | --- | --- | --- | --- | --- | --- | --- |
| 1 | (Constant) | -2.028 | 0.040 |  | -50.803 | 0.000000 | -2.106 | -1.949 | .258 |
|  | Age | 0.016 | 0.001 | 0.208 | 28.928 | 0.000000 | 0.015 | 0.018 |  |
|  | Income | 0.000 | 0.000 | 0.032 | 4.765 | 0.000002 | 0.000 | 0.000 |  |
|  | Wave 1 Envhabit7 | 0.512 | 0.007 | 0.547 | 77.508 | 0.000000 | 0.499 | 0.525 |  |
| 2 | (Constant) | -2.015 | 0.042 |  | -48.512 | 0.000000 | -2.096 | -1.933 | .258 |
|  | Age | 0.016 | 0.001 | 0.206 | 27.39 | 0.000000 | 0.015 | 0.017 |  |
|  | Income | 0.000 | 0.000 | 0.032 | 4.738 | 0.000002 | 0.000 | 0.000 |  |
|  | Wave 1 Envhabit7 | 0.512 | 0.007 | 0.547 | 77.488 | 0.000000 | 0.499 | 0.525 |  |
|  | Newborn | -0.037 | 0.033 | -0.008 | -1.118 | 0.263736 | -0.103 | 0.028 |  |

| **Model** | **Dependent Variable: Change in envhabit8** | **B** | **SE** | **β** | **t** | **p** | **Lower 95%CI** | **Upper 95%CI** | **R^2^** |
| --- | --- | --- | --- | --- | --- | --- | --- | --- | --- |
| 1 | (Constant) | -1.705 | 0.033 |  | -52.357 | 0.000000 | -1.769 | -1.641 | .223 |
|  | Age | 0.001 | 0.000 | 0.011 | 1.531 | 0.125693 | 0.000 | 0.002 |  |
|  | Income | 0.000 | 0.000 | -0.018 | -2.589 | 0.009645 | 0.000 | 0.000 |  |
|  | Wave 1 Envhabit8 | 0.405 | 0.006 | 0.483 | 69.878 | 0.000000 | 0.394 | 0.416 |  |
| 2 | (Constant) | -1.669 | 0.034 |  | -49.105 | 0.000000 | -1.735 | -1.602 | .223 |
|  | Age | 0.000 | 0.001 | 0.003 | 0.372 | 0.710127 | -0.001 | 0.001 |  |
|  | Income | 0.000 | 0.000 | -0.019 | -2.681 | 0.007358 | 0.000 | 0.000 |  |
|  | Wave 1 Envhabit8 | 0.405 | 0.006 | 0.483 | 69.919 | 0.000000 | 0.394 | 0.416 |  |
|  | Newborn | -0.111 | 0.030 | -0.027 | -3.691 | 0.000224 | -0.169 | -0.052 |  |

| **Model** | **Dependent Variable: Change in envhabit9** | **B** | **SE** | **β** | **t** | **p** | **Lower 95%CI** | **Upper 95%CI** | **R^2^** |
| --- | --- | --- | --- | --- | --- | --- | --- | --- | --- |
| 1 | (Constant) | -1.428 | 0.035 |  | -40.919 | 0.000000 | -1.497 | -1.360 | .275 |
|  | Age | -0.006 | 0.001 | -0.075 | -11.093 | 0.000000 | -0.007 | -0.005 |  |
|  | Income | 0.000 | 0.000 | -0.005 | -0.71 | 0.477752 | 0.000 | 0.000 |  |
|  | Wave 1 Envhabit9 | 0.543 | 0.007 | 0.528 | 79.387 | 0.000000 | 0.530 | 0.557 |  |
| 2 | (Constant) | -1.439 | 0.037 |  | -39.378 | 0.000000 | -1.511 | -1.368 | .275 |
|  | Age | -0.006 | 0.001 | -0.073 | -10.299 | 0.000000 | -0.007 | -0.005 |  |
|  | Income | 0.000 | 0.000 | -0.005 | -0.683 | 0.494655 | 0.000 | 0.000 |  |
|  | Wave 1 Envhabit9 | 0.543 | 0.007 | 0.527 | 79.344 | 0.000000 | 0.530 | 0.557 |  |
|  | Newborn | 0.035 | 0.034 | 0.007 | 1.033 | 0.301578 | -0.031 | 0.101 |  |

| **Model** | **Dependent Variable: Change in envhabit10** | **B** | **SE** | **β** | **t** | **p** | **Lower 95%CI** | **Upper 95%CI** | **R^2^** |
| --- | --- | --- | --- | --- | --- | --- | --- | --- | --- |
| 1 | (Constant) | -2.709 | 0.043 |  | -62.446 | 0.000000 | -2.794 | -2.624 | .394 |
|  | Age | -0.006 | 0.001 | -0.072 | -9.474 | 0.000000 | -0.007 | -0.005 |  |
|  | Income | 0.000 | 0.000 | -0.010 | -1.412 | 0.158073 | 0.000 | 0.000 |  |
|  | Wave 1 Envhabit10 | 0.707 | 0.008 | 0.639 | 85.695 | 0.000000 | 0.691 | 0.723 |  |
| 2 | (Constant) | -2.664 | 0.045 |  | -59.5 | 0.000000 | -2.752 | -2.576 | .395 |
|  | Age | -0.007 | 0.001 | -0.081 | -10.247 | 0.000000 | -0.008 | -0.005 |  |
|  | Income | 0.000 | 0.000 | -0.011 | -1.488 | 0.136817 | 0.000 | 0.000 |  |
|  | Wave 1 Envhabit10 | 0.708 | 0.008 | 0.639 | 85.835 | 0.000000 | 0.692 | 0.724 |  |
|  | Newborn | -0.149 | 0.037 | -0.031 | -4.049 | 0.000052 | -0.222 | -0.077 |  |

| **Model** | **Dependent Variable: Change in envhabit11** | **B** | **SE** | **β** | **t** | **p** | **Lower 95%CI** | **Upper 95%CI** | **R^2^** |
| --- | --- | --- | --- | --- | --- | --- | --- | --- | --- |
| 1 | (Constant) | -3.390 | 0.051 |  | -66.329 | 0.000000 | -3.490 | -3.289 | .493 |
|  | Age | -0.003 | 0.001 | -0.036 | -4.509 | 0.000007 | -0.004 | -0.002 |  |
|  | Income | 0.000 | 0.000 | -0.006 | -0.725 | 0.468407 | 0.000 | 0.000 |  |
|  | Wave 1 Envhabit11 | 0.766 | 0.009 | 0.703 | 88.428 | 0.000000 | 0.749 | 0.783 |  |
| 2 | (Constant) | -3.391 | 0.053 |  | -64.462 | 0.000000 | -3.494 | -3.288 | .493 |
|  | Age | -0.003 | 0.001 | -0.036 | -4.293 | 0.000018 | -0.004 | -0.002 |  |
|  | Income | 0.000 | 0.000 | -0.006 | -0.724 | 0.469018 | 0.000 | 0.000 |  |
|  | Wave 1 Envhabit11 | 0.766 | 0.009 | 0.703 | 88.409 | 0.000000 | 0.749 | 0.783 |  |
|  | Newborn | 0.004 | 0.041 | 0.001 | 0.099 | 0.921074 | -0.077 | 0.085 |  |

## New Parent Status

| **Model** | **Dependent Variable: Change in ftst** | **B** | **SE** | **β** | **t** | **p** | **Lower 95%CI** | **Upper 95%CI** | **R^2^** |
| --- | --- | --- | --- | --- | --- | --- | --- | --- | --- |
| 1 | (Constant) | 1.019 | 0.017 |  | 60.706 | 0.000000 | 0.986 | 1.052 | .332 |
|  | Age | -0.003 | 0.000 | -0.083 | -13.184 | 0.000000 | -0.003 | -0.002 |  |
|  | Income | 0.000 | 0.000 | 0.012 | 2.003 | 0.045163 | 0.000 | 0.000 |  |
|  | Wave 1 Ftst | -0.650 | 0.007 | -0.584 | -94.55 | 0.000000 | -0.664 | -0.637 |  |
| 2 | (Constant) | 1.015 | 0.017 |  | 59.532 | 0.000000 | 0.982 | 1.049 | .332 |
|  | Age | -0.003 | 0.000 | -0.081 | -12.655 | 0.000000 | -0.003 | -0.002 |  |
|  | Income | 0.000 | 0.000 | 0.012 | 2.005 | 0.044936 | 0.000 | 0.000 |  |
|  | Wave 1 Ftst | -0.650 | 0.007 | -0.584 | -94.547 | 0.000000 | -0.664 | -0.637 |  |
|  | New Parent | 0.024 | 0.020 | 0.007 | 1.19 | 0.234218 | -0.016 | 0.064 |  |

| **Model** | **Dependent Variable: Change in crlf** | **B** | **SE** | **β** | **t** | **p** | **Lower 95%CI** | **Upper 95%CI** | **R^2^** |
| --- | --- | --- | --- | --- | --- | --- | --- | --- | --- |
| 1 | (Constant) | 1.454 | 0.025 |  | 58.844 | 0.000000 | 1.406 | 1.503 | .302 |
|  | Age | 0.005 | 0.000 | 0.103 | 15.723 | 0.000000 | 0.005 | 0.006 |  |
|  | Income | 0.000 | 0.000 | 0.002 | 0.369 | 0.711800 | 0.000 | 0.000 |  |
|  | Wave 1 crlf | -0.623 | 0.007 | -0.562 | -88.074 | 0.000000 | -0.637 | -0.609 |  |
| 2 | (Constant) | 1.465 | 0.025 |  | 58.279 | 0.000000 | 1.416 | 1.514 | .302 |
|  | Age | 0.005 | 0.000 | 0.099 | 14.921 | 0.000000 | 0.005 | 0.006 |  |
|  | Income | 0.000 | 0.000 | 0.002 | 0.364 | 0.715773 | 0.000 | 0.000 |  |
|  | Wave 1 crlf | -0.623 | 0.007 | -0.563 | -88.09 | 0.000000 | -0.637 | -0.609 |  |
|  | New Parent | -0.072 | 0.031 | -0.015 | -2.335 | 0.019533 | -0.132 | -0.012 |  |

| **Model** | **Dependent Variable: Change in grn** | **B** | **SE** | **β** | **t** | **p** | **Lower 95%CI** | **Upper 95%CI** | **R^2^** |
| --- | --- | --- | --- | --- | --- | --- | --- | --- | --- |
| 1 | (Constant) | 1.671 | 0.023 |  | 71.308 | 0.000000 | 1.625 | 1.717 | .334 |
|  | Age | -0.002 | 0.000 | -0.044 | -6.995 | 0.000000 | -0.003 | -0.001 |  |
|  | Income | 0.000 | 0.000 | 0.039 | 6.2 | 0.000000 | 0.000 | 0.000 |  |
|  | Wave 1 grn | -0.638 | 0.007 | -0.586 | -93.864 | 0.000000 | -0.652 | -0.625 |  |
| 2 | (Constant) | 1.674 | 0.024 |  | 70.572 | 0.000000 | 1.628 | 1.721 | .334 |
|  | Age | -0.002 | 0.000 | -0.045 | -7 | 0.000000 | -0.003 | -0.001 |  |
|  | Income | 0.000 | 0.000 | 0.039 | 6.198 | 0.000000 | 0.000 | 0.000 |  |
|  | Wave 1 grn | -0.638 | 0.007 | -0.586 | -93.853 | 0.000000 | -0.652 | -0.625 |  |
|  | New Parent | -0.019 | 0.025 | -0.005 | -0.755 | 0.450557 | -0.069 | 0.031 |  |

| **Model** | **Dependent Variable: Change in envhabit1** | **B** | **SE** | **β** | **t** | **p** | **Lower 95%CI** | **Upper 95%CI** | **R^2^** |
| --- | --- | --- | --- | --- | --- | --- | --- | --- | --- |
| 1 | (Constant) | 1.276 | 0.048 |  | 26.806 | 0.000000 | 1.183 | 1.370 | .236 |
|  | Age | 0.008 | 0.001 | 0.077 | 11.373 | 0.000000 | 0.007 | 0.009 |  |
|  | Income | 0.000 | 0.000 | -0.026 | -3.856 | 0.000116 | 0.000 | 0.000 |  |
|  | Wave 1 Envhabit1 | -0.539 | 0.007 | -0.488 | -73.904 | 0.000000 | -0.553 | -0.524 |  |
| 2 | (Constant) | 1.284 | 0.049 |  | 26.343 | 0.000000 | 1.188 | 1.379 | .236 |
|  | Age | 0.008 | 0.001 | 0.076 | 10.98 | 0.000000 | 0.006 | 0.009 |  |
|  | Income | 0.000 | 0.000 | -0.026 | -3.858 | 0.000115 | 0.000 | 0.000 |  |
|  | Wave 1 Envhabit1 | -0.539 | 0.007 | -0.489 | -73.89 | 0.000000 | -0.553 | -0.524 |  |
|  | New Parent | -0.044 | 0.063 | -0.005 | -0.688 | 0.491243 | -0.167 | 0.080 |  |

| **Model** | **Dependent Variable: Change in envhabit2** | **B** | **SE** | **β** | **t** | **p** | **Lower 95%CI** | **Upper 95%CI** | **R^2^** |
| --- | --- | --- | --- | --- | --- | --- | --- | --- | --- |
| 1 | (Constant) | -1.237 | 0.025 |  | -50.012 | 0.000000 | -1.285 | -1.188 | .398 |
|  | Age | 0.003 | 0.000 | 0.053 | 9.001 | 0.000000 | 0.003 | 0.004 |  |
|  | Income | 0.000 | 0.000 | -0.034 | -5.758 | 0.000000 | 0.000 | 0.000 |  |
|  | Wave 1 Envhabit2 | 0.730 | 0.007 | 0.638 | 109.283 | 0.000000 | 0.717 | 0.743 |  |
| 2 | (Constant) | -1.219 | 0.025 |  | -48.376 | 0.000000 | -1.269 | -1.170 | .398 |
|  | Age | 0.003 | 0.000 | 0.049 | 8.064 | 0.000000 | 0.002 | 0.004 |  |
|  | Income | 0.000 | 0.000 | -0.034 | -5.774 | 0.000000 | 0.000 | 0.000 |  |
|  | Wave 1 Envhabit2 | 0.730 | 0.007 | 0.638 | 109.342 | 0.000000 | 0.717 | 0.743 |  |
|  | New Parent | -0.120 | 0.033 | -0.021 | -3.603 | 0.000315 | -0.186 | -0.055 |  |

| **Model** | **Dependent Variable: Change in envhabit3** | **B** | **SE** | **β** | **t** | **p** | **Lower 95%CI** | **Upper 95%CI** | **R^2^** |
| --- | --- | --- | --- | --- | --- | --- | --- | --- | --- |
| 1 | (Constant) | 1.335 | 0.039 |  | 34.027 | 0.000000 | 1.258 | 1.411 | .240 |
|  | Age | 0.005 | 0.001 | 0.052 | 7.851 | 0.000000 | 0.004 | 0.006 |  |
|  | Income | 0.000 | 0.000 | 0.023 | 3.449 | 0.000564 | 0.000 | 0.000 |  |
|  | Wave 1 Envhabit3 | -0.459 | 0.006 | -0.489 | -75.102 | 0.000000 | -0.471 | -0.447 |  |
| 2 | (Constant) | 1.346 | 0.040 |  | 33.537 | 0.000000 | 1.268 | 1.425 | .240 |
|  | Age | 0.005 | 0.001 | 0.050 | 7.391 | 0.000000 | 0.003 | 0.006 |  |
|  | Income | 0.000 | 0.000 | 0.023 | 3.445 | 0.000572 | 0.000 | 0.000 |  |
|  | Wave 1 Envhabit3 | -0.459 | 0.006 | -0.490 | -75.115 | 0.000000 | -0.471 | -0.447 |  |
|  | New Parent | -0.075 | 0.054 | -0.009 | -1.382 | 0.167010 | -0.181 | 0.031 |  |

| **Model** | **Dependent Variable: Change in envhabit4** | **B** | **SE** | **β** | **t** | **p** | **Lower 95%CI** | **Upper 95%CI** | **R^2^** |
| --- | --- | --- | --- | --- | --- | --- | --- | --- | --- |
| 1 | (Constant) | -1.549 | 0.034 |  | -45.55 | 0.000000 | -1.616 | -1.483 | .334 |
|  | Age | -0.001 | 0.001 | -0.014 | -2.226 | 0.026021 | -0.002 | 0.000 |  |
|  | Income | 0.000 | 0.000 | -0.003 | -0.553 | 0.580469 | 0.000 | 0.000 |  |
|  | Wave 1 Envhabit4 | 0.644 | 0.007 | 0.577 | 94.847 | 0.000000 | 0.631 | 0.657 |  |
| 2 | (Constant) | -1.530 | 0.035 |  | -44.194 | 0.000000 | -1.598 | -1.463 | .334 |
|  | Age | -0.001 | 0.001 | -0.018 | -2.79 | 0.005276 | -0.002 | 0.000 |  |
|  | Income | 0.000 | 0.000 | -0.004 | -0.564 | 0.572707 | 0.000 | 0.000 |  |
|  | Wave 1 Envhabit4 | 0.644 | 0.007 | 0.578 | 94.908 | 0.000000 | 0.631 | 0.658 |  |
|  | New Parent | -0.134 | 0.046 | -0.018 | -2.923 | 0.003467 | -0.225 | -0.044 |  |

| **Model** | **Dependent Variable: Change in envhabit5** | **B** | **SE** | **β** | **t** | **p** | **Lower 95%CI** | **Upper 95%CI** | **R^2^** |
| --- | --- | --- | --- | --- | --- | --- | --- | --- | --- |
| 1 | (Constant) | -2.766 | 0.037 |  | -75.333 | 0.000000 | -2.838 | -2.694 | .341 |
|  | Age | 0.000 | 0.000 | 0.000 | 0.002 | 0.998725 | -0.001 | 0.001 |  |
|  | Income | 0.000 | 0.000 | 0.015 | 2.476 | 0.013287 | 0.000 | 0.000 |  |
|  | Wave 1 Envhabit5 | 0.625 | 0.007 | 0.584 | 95.184 | 0.000000 | 0.612 | 0.638 |  |
| 2 | (Constant) | -2.772 | 0.037 |  | -74.721 | 0.000000 | -2.845 | -2.699 | .341 |
|  | Age | 0.000 | 0.000 | 0.001 | 0.221 | 0.825242 | -0.001 | 0.001 |  |
|  | Income | 0.000 | 0.000 | 0.016 | 2.479 | 0.013177 | 0.000 | 0.000 |  |
|  | Wave 1 Envhabit5 | 0.625 | 0.007 | 0.584 | 95.188 | 0.000000 | 0.612 | 0.638 |  |
|  | New Parent | 0.035 | 0.034 | 0.007 | 1.043 | 0.296899 | -0.031 | 0.102 |  |

| **Model** | **Dependent Variable: Change in envhabit6** | **B** | **SE** | **β** | **t** | **p** | **Lower 95%CI** | **Upper 95%CI** | **R^2^** |
| --- | --- | --- | --- | --- | --- | --- | --- | --- | --- |
| 1 | (Constant) | -2.420 | 0.041 |  | -59.735 | 0.000000 | -2.499 | -2.340 | .329 |
|  | Age | 0.002 | 0.001 | 0.019 | 2.909 | 0.003626 | 0.001 | 0.003 |  |
|  | Income | 0.000 | 0.000 | 0.016 | 2.503 | 0.012309 | 0.000 | 0.000 |  |
|  | Wave 1 Envhabit6 | 0.628 | 0.007 | 0.575 | 91.058 | 0.000000 | 0.615 | 0.642 |  |
| 2 | (Constant) | -2.423 | 0.041 |  | -58.877 | 0.000000 | -2.504 | -2.342 | .329 |
|  | Age | 0.002 | 0.001 | 0.019 | 2.943 | 0.003254 | 0.001 | 0.003 |  |
|  | Income | 0.000 | 0.000 | 0.016 | 2.503 | 0.012331 | 0.000 | 0.000 |  |
|  | Wave 1 Envhabit6 | 0.628 | 0.007 | 0.575 | 91.055 | 0.000000 | 0.615 | 0.642 |  |
|  | New Parent | 0.022 | 0.047 | 0.003 | 0.472 | 0.637000 | -0.071 | 0.115 |  |

| **Model** | **Dependent Variable: Change in envhabit7** | **B** | **SE** | **β** | **t** | **p** | **Lower 95%CI** | **Upper 95%CI** | **R^2^** |
| --- | --- | --- | --- | --- | --- | --- | --- | --- | --- |
| 1 | (Constant) | -2.028 | 0.040 |  | -50.803 | 0.000000 | -2.106 | -1.949 | .258 |
|  | Age | 0.016 | 0.001 | 0.208 | 28.928 | 0.000000 | 0.015 | 0.018 |  |
|  | Income | 0.000 | 0.000 | 0.032 | 4.765 | 0.000002 | 0.000 | 0.000 |  |
|  | Wave 1 Envhabit7 | 0.512 | 0.007 | 0.547 | 77.508 | 0.000000 | 0.499 | 0.525 |  |
| 2 | (Constant) | -2.025 | 0.040 |  | -50.033 | 0.000000 | -2.105 | -1.946 | .258 |
|  | Age | 0.016 | 0.001 | 0.208 | 28.335 | 0.000000 | 0.015 | 0.018 |  |
|  | Income | 0.000 | 0.000 | 0.032 | 4.765 | 0.000002 | 0.000 | 0.000 |  |
|  | Wave 1 Envhabit7 | 0.512 | 0.007 | 0.547 | 77.498 | 0.000000 | 0.499 | 0.525 |  |
|  | New Parent | -0.016 | 0.048 | -0.002 | -0.34 | 0.733729 | -0.110 | 0.078 |  |

| **Model** | **Dependent Variable: Change in envhabit8** | **B** | **SE** | **β** | **t** | **p** | **Lower 95%CI** | **Upper 95%CI** | **R^2^** |
| --- | --- | --- | --- | --- | --- | --- | --- | --- | --- |
| 1 | (Constant) | -1.705 | 0.033 |  | -52.357 | 0.000000 | -1.769 | -1.641 | .233 |
|  | Age | 0.001 | 0.000 | 0.011 | 1.531 | 0.125693 | 0.000 | 0.002 |  |
|  | Income | 0.000 | 0.000 | -0.018 | -2.589 | 0.009645 | 0.000 | 0.000 |  |
|  | Wave 1 Envhabit8 | 0.405 | 0.006 | 0.483 | 69.878 | 0.000000 | 0.394 | 0.416 |  |
| 2 | (Constant) | -1.686 | 0.033 |  | -50.651 | 0.000000 | -1.751 | -1.621 | .233 |
|  | Age | 0.000 | 0.000 | 0.007 | 0.92 | 0.357404 | -0.001 | 0.001 |  |
|  | Income | 0.000 | 0.000 | -0.018 | -2.594 | 0.009487 | 0.000 | 0.000 |  |
|  | Wave 1 Envhabit8 | 0.405 | 0.006 | 0.483 | 69.834 | 0.000000 | 0.393 | 0.416 |  |
|  | New Parent | -0.114 | 0.042 | -0.019 | -2.714 | 0.006660 | -0.197 | -0.032 |  |

| **Model** | **Dependent Variable: Change in envhabit9** | **B** | **SE** | **β** | **t** | **p** | **Lower 95%CI** | **Upper 95%CI** | **R^2^** |
| --- | --- | --- | --- | --- | --- | --- | --- | --- | --- |
| 1 | (Constant) | -1.428 | 0.035 |  | -40.919 | 0.000000 | -1.497 | -1.360 | .275 |
|  | Age | -0.006 | 0.001 | -0.075 | -11.093 | 0.000000 | -0.007 | -0.005 |  |
|  | Income | 0.000 | 0.000 | -0.005 | -0.71 | 0.477752 | 0.000 | 0.000 |  |
|  | Wave 1 Envhabit9 | 0.543 | 0.007 | 0.528 | 79.387 | 0.000000 | 0.530 | 0.557 |  |
| 2 | (Constant) | -1.435 | 0.036 |  | -40.312 | 0.000000 | -1.505 | -1.365 | .275 |
|  | Age | -0.006 | 0.001 | -0.073 | -10.641 | 0.000000 | -0.007 | -0.005 |  |
|  | Income | 0.000 | 0.000 | -0.005 | -0.707 | 0.479730 | 0.000 | 0.000 |  |
|  | Wave 1 Envhabit9 | 0.543 | 0.007 | 0.527 | 79.321 | 0.000000 | 0.530 | 0.557 |  |
|  | New Parent | 0.045 | 0.048 | 0.006 | 0.943 | 0.345839 | -0.049 | 0.139 |  |

| **Model** | **Dependent Variable: Change in envhabit10** | **B** | **SE** | **β** | **t** | **p** | **Lower 95%CI** | **Upper 95%CI** | **R^2^** |
| --- | --- | --- | --- | --- | --- | --- | --- | --- | --- |
| 1 | (Constant) | -2.709 | 0.043 |  | -62.446 | 0.000000 | -2.794 | -2.624 | .394 |
|  | Age | -0.006 | 0.001 | -0.072 | -9.474 | 0.000000 | -0.007 | -0.005 |  |
|  | Income | 0.000 | 0.000 | -0.010 | -1.412 | 0.158073 | 0.000 | 0.000 |  |
|  | Wave 1 Envhabit10 | 0.707 | 0.008 | 0.639 | 85.695 | 0.000000 | 0.691 | 0.723 |  |
| 2 | (Constant) | -2.708 | 0.044 |  | -61.312 | 0.000000 | -2.794 | -2.621 | .394 |
|  | Age | -0.006 | 0.001 | -0.072 | -9.303 | 0.000000 | -0.007 | -0.005 |  |
|  | Income | 0.000 | 0.000 | -0.010 | -1.413 | 0.157789 | 0.000 | 0.000 |  |
|  | Wave 1 Envhabit10 | 0.707 | 0.008 | 0.639 | 85.692 | 0.000000 | 0.691 | 0.723 |  |
|  | New Parent | -0.010 | 0.051 | -0.001 | -0.198 | 0.843079 | -0.110 | 0.090 |  |

| **Model** | **Dependent Variable: Change in envhabit11** | **B** | **SE** | **β** | **t** | **p** | **Lower 95%CI** | **Upper 95%CI** | **R^2^** |
| --- | --- | --- | --- | --- | --- | --- | --- | --- | --- |
| 1 | (Constant) | -3.390 | 0.051 |  | -66.329 | 0.000000 | -3.490 | -3.289 | .493 |
|  | Age | -0.003 | 0.001 | -0.036 | -4.509 | 0.000007 | -0.004 | -0.002 |  |
|  | Income | 0.000 | 0.000 | -0.006 | -0.725 | 0.468407 | 0.000 | 0.000 |  |
|  | Wave 1 Envhabit11 | 0.766 | 0.009 | 0.703 | 88.428 | 0.000000 | 0.749 | 0.783 |  |
| 2 | (Constant) | -3.393 | 0.052 |  | -65.47 | 0.000000 | -3.495 | -3.291 | .493 |
|  | Age | -0.003 | 0.001 | -0.036 | -4.327 | 0.000015 | -0.004 | -0.002 |  |
|  | Income | 0.000 | 0.000 | -0.006 | -0.73 | 0.465227 | 0.000 | 0.000 |  |
|  | Wave 1 Envhabit11 | 0.766 | 0.009 | 0.703 | 88.421 | 0.000000 | 0.749 | 0.783 |  |
|  | New Parent | 0.023 | 0.057 | 0.003 | 0.412 | 0.680445 | -0.088 | 0.135 |  |

## New Eco-Parent

| **Model** | **Dependent Variable: Change in ftst** | **B** | **SE** | **β** | **t** | **p** | **Lower 95%CI** | **Upper 95%CI** | **R^2^** |
| --- | --- | --- | --- | --- | --- | --- | --- | --- | --- |
| 1 | (Constant) | 1.019 | 0.017 |  | 60.582 | 0.000000 | 0.986 | 1.052 | .332 |
|  | Age | -0.003 | 0.000 | -0.083 | -13.195 | 0.000000 | -0.003 | -0.003 |  |
|  | Income | 0.000 | 0.000 | 0.013 | 2.01 | 0.044399 | 0.000 | 0.000 |  |
|  | Wave 1 Ftst | -0.650 | 0.007 | -0.584 | -94.416 | 0.000000 | -0.664 | -0.637 |  |
| 2 | (Constant) | 1.013 | 0.017 |  | 59.866 | 0.000000 | 0.980 | 1.046 | .332 |
|  | Age | -0.003 | 0.000 | -0.080 | -12.556 | 0.000000 | -0.003 | -0.002 |  |
|  | Income | 0.000 | 0.000 | 0.012 | 1.947 | 0.051541 | 0.000 | 0.000 |  |
|  | Wave 1 Ftst | -0.651 | 0.007 | -0.584 | -94.501 | 0.000000 | -0.664 | -0.637 |  |
|  | New Eco-Parent | 0.093 | 0.026 | 0.022 | 3.605 | 0.000313 | 0.042 | 0.143 |  |

| **Model** | **Dependent Variable: Change in crlf** | **B** | **SE** | **β** | **t** | **p** | **Lower 95%CI** | **Upper 95%CI** | **R^2^** |
| --- | --- | --- | --- | --- | --- | --- | --- | --- | --- |
| 1 | (Constant) | 1.454 | 0.025 |  | 58.727 | 0.000000 | 1.406 | 1.503 | .302 |
|  | Age | 0.005 | 0.000 | 0.102 | 15.682 | 0.000000 | 0.005 | 0.006 |  |
|  | Income | 0.000 | 0.000 | 0.002 | 0.35 | 0.726001 | 0.000 | 0.000 |  |
|  | Wave 1 crlf | -0.623 | 0.007 | -0.562 | -87.919 | 0.000000 | -0.637 | -0.609 |  |
| 2 | (Constant) | 1.456 | 0.025 |  | 58.408 | 0.000000 | 1.407 | 1.505 | .302 |
|  | Age | 0.005 | 0.000 | 0.102 | 15.408 | 0.000000 | 0.005 | 0.006 |  |
|  | Income | 0.000 | 0.000 | 0.002 | 0.364 | 0.716095 | 0.000 | 0.000 |  |
|  | Wave 1 crlf | -0.623 | 0.007 | -0.562 | -87.901 | 0.000000 | -0.637 | -0.609 |  |
|  | New Eco-Parent | -0.028 | 0.039 | -0.005 | -0.73 | 0.465427 | -0.105 | 0.048 |  |

| **Model** | **Dependent Variable: Change in grn** | **B** | **SE** | **β** | **t** | **p** | **Lower 95%CI** | **Upper 95%CI** | **R^2^** |
| --- | --- | --- | --- | --- | --- | --- | --- | --- | --- |
| 1 | (Constant) | 1.674 | 0.024 |  | 71.212 | 0.000000 | 1.628 | 1.720 | .334 |
|  | Age | -0.002 | 0.000 | -0.045 | -7.098 | 0.000000 | -0.003 | -0.001 |  |
|  | Income | 0.000 | 0.000 | 0.039 | 6.134 | 0.000000 | 0.000 | 0.000 |  |
|  | Wave 1 grn | -0.639 | 0.007 | -0.586 | -93.777 | 0.000000 | -0.652 | -0.625 |  |
| 2 | (Constant) | 1.671 | 0.024 |  | 70.914 | 0.000000 | 1.624 | 1.717 | .335 |
|  | Age | -0.002 | 0.000 | -0.043 | -6.694 | 0.000000 | -0.002 | -0.001 |  |
|  | Income | 0.000 | 0.000 | 0.039 | 6.114 | 0.000000 | 0.000 | 0.000 |  |
|  | Wave 1 grn | -0.640 | 0.007 | -0.587 | -93.751 | 0.000000 | -0.653 | -0.626 |  |
|  | New Eco-Parent | 0.075 | 0.032 | 0.015 | 2.34 | 0.019301 | 0.012 | 0.138 |  |

| **Model** | **Dependent Variable: Change in envhabit1** | **B** | **SE** | **β** | **T** | **p** | **Lower 95%CI** | **Upper 95%CI** | **R^2^** |
| --- | --- | --- | --- | --- | --- | --- | --- | --- | --- |
| 1 | (Constant) | 1.274 | 0.048 |  | 26.693 | 0.000000 | 1.180 | 1.367 | .236 |
|  | Age | 0.008 | 0.001 | 0.077 | 11.396 | 0.000000 | 0.007 | 0.009 |  |
|  | Income | 0.000 | 0.000 | -0.026 | -3.934 | 0.000084 | 0.000 | 0.000 |  |
|  | Wave 1 Envhabit1 | -0.538 | 0.007 | -0.488 | -73.76 | 0.000000 | -0.552 | -0.524 |  |
| 2 | (Constant) | 1.280 | 0.048 |  | 26.543 | 0.000000 | 1.185 | 1.374 | .236 |
|  | Age | 0.008 | 0.001 | 0.076 | 11.144 | 0.000000 | 0.006 | 0.009 |  |
|  | Income | 0.000 | 0.000 | -0.026 | -3.918 | 0.000090 | 0.000 | 0.000 |  |
|  | Wave 1 Envhabit1 | -0.538 | 0.007 | -0.488 | -73.764 | 0.000000 | -0.552 | -0.524 |  |
|  | New Eco-Parent | -0.069 | 0.080 | -0.006 | -0.868 | 0.385402 | -0.226 | 0.087 |  |

| **Model** | **Dependent Variable: Change in envhabit2** | **B** | **SE** | **β** | **T** | **p** | **Lower 95%CI** | **Upper 95%CI** | **R^2^** |
| --- | --- | --- | --- | --- | --- | --- | --- | --- | --- |
| 1 | (Constant) | -1.236 | 0.025 |  | -49.851 | 0.000000 | -1.284 | -1.187 | .397 |
|  | Age | 0.003 | 0.000 | 0.053 | 8.927 | 0.000000 | 0.003 | 0.004 |  |
|  | Income | 0.000 | 0.000 | -0.034 | -5.669 | 0.000000 | 0.000 | 0.000 |  |
|  | Wave 1 Envhabit2 | 0.729 | 0.007 | 0.637 | 108.933 | 0.000000 | 0.716 | 0.743 |  |
| 2 | (Constant) | -1.231 | 0.025 |  | -49.149 | 0.000000 | -1.280 | -1.182 | .397 |
|  | Age | 0.003 | 0.000 | 0.052 | 8.626 | 0.000000 | 0.002 | 0.004 |  |
|  | Income | 0.000 | 0.000 | -0.033 | -5.643 | 0.000000 | 0.000 | 0.000 |  |
|  | Wave 1 Envhabit2 | 0.729 | 0.007 | 0.637 | 108.906 | 0.000000 | 0.716 | 0.742 |  |
|  | New Eco-Parent | -0.059 | 0.042 | -0.008 | -1.385 | 0.166090 | -0.142 | 0.024 |  |

| **Model** | **Dependent Variable: Change in envhabit3** | **B** | **SE** | **β** | **t** | **p** | **Lower 95%CI** | **Upper 95%CI** | **R^2^** |
| --- | --- | --- | --- | --- | --- | --- | --- | --- | --- |
| 1 | (Constant) | 1.328 | 0.039 |  | 33.772 | 0.000000 | 1.250 | 1.405 | .240 |
|  | Age | 0.005 | 0.001 | 0.053 | 7.975 | 0.000000 | 0.004 | 0.006 |  |
|  | Income | 0.000 | 0.000 | 0.023 | 3.389 | 0.000704 | 0.000 | 0.000 |  |
|  | Wave 1 Envhabit3 | -0.458 | 0.006 | -0.489 | -74.908 | 0.000000 | -0.470 | -0.446 |  |
| 2 | (Constant) | 1.330 | 0.040 |  | 33.522 | 0.000000 | 1.252 | 1.408 | .240 |
|  | Age | 0.005 | 0.001 | 0.053 | 7.818 | 0.000000 | 0.004 | 0.006 |  |
|  | Income | 0.000 | 0.000 | 0.023 | 3.397 | 0.000684 | 0.000 | 0.000 |  |
|  | Wave 1 Envhabit3 | -0.458 | 0.006 | -0.489 | -74.901 | 0.000000 | -0.470 | -0.446 |  |
|  | New Eco-Parent | -0.033 | 0.069 | -0.003 | -0.475 | 0.634661 | -0.167 | 0.102 |  |

| **Model** | **Dependent Variable: Change in envhabit4** | **B** | **SE** | **β** | **t** | **p** | **Lower 95%CI** | **Upper 95%CI** | **R^2^** |
| --- | --- | --- | --- | --- | --- | --- | --- | --- | --- |
| 1 | (Constant) | -1.547 | 0.034 |  | -45.369 | 0.000000 | -1.614 | -1.481 | .333 |
|  | Age | -0.001 | 0.001 | -0.014 | -2.283 | 0.022445 | -0.002 | 0.000 |  |
|  | Income | 0.000 | 0.000 | -0.004 | -0.563 | 0.573612 | 0.000 | 0.000 |  |
|  | Wave 1 Envhabit4 | 0.644 | 0.007 | 0.577 | 94.643 | 0.000000 | 0.631 | 0.657 |  |
| 2 | (Constant) | -1.547 | 0.034 |  | -44.948 | 0.000000 | -1.615 | -1.480 | .333 |
|  | Age | -0.001 | 0.001 | -0.014 | -2.267 | 0.023375 | -0.002 | 0.000 |  |
|  | Income | 0.000 | 0.000 | -0.003 | -0.562 | 0.574448 | 0.000 | 0.000 |  |
|  | Wave 1 Envhabit4 | 0.644 | 0.007 | 0.577 | 94.64 | 0.000000 | 0.631 | 0.657 |  |
|  | New Eco-Parent | -0.004 | 0.058 | 0.000 | -0.064 | 0.949068 | -0.118 | 0.111 |  |

| **Model** | **Dependent Variable: Change in envhabit5** | **B** | **SE** | **β** | **t** | **p** | **Lower 95%CI** | **Upper 95%CI** | **R^2^** |
| --- | --- | --- | --- | --- | --- | --- | --- | --- | --- |
| 1 | (Constant) | -2.773 | 0.037 |  | -75.331 | 0.000000 | -2.845 | -2.700 | .342 |
|  | Age | 0.000 | 0.000 | 0.001 | 0.12 | 0.904851 | -0.001 | 0.001 |  |
|  | Income | 0.000 | 0.000 | 0.016 | 2.578 | 0.009945 | 0.000 | 0.000 |  |
|  | Wave 1 Envhabit5 | 0.626 | 0.007 | 0.585 | 95.146 | 0.000000 | 0.613 | 0.639 |  |
| 2 | (Constant) | -2.779 | 0.037 |  | -75.036 | 0.000000 | -2.851 | -2.706 | .342 |
|  | Age | 0.000 | 0.000 | 0.002 | 0.34 | 0.733747 | -0.001 | 0.001 |  |
|  | Income | 0.000 | 0.000 | 0.016 | 2.55 | 0.010776 | 0.000 | 0.000 |  |
|  | Wave 1 Envhabit5 | 0.626 | 0.007 | 0.585 | 95.16 | 0.000000 | 0.613 | 0.639 |  |
|  | New Eco-Parent | 0.065 | 0.043 | 0.009 | 1.502 | 0.133091 | -0.020 | 0.149 |  |

| **Model** | **Dependent Variable: Change in envhabit6** | **B** | **SE** | **β** | **t** | **p** | **Lower 95%CI** | **Upper 95%CI** | **R^2^** |
| --- | --- | --- | --- | --- | --- | --- | --- | --- | --- |
| 1 | (Constant) | -2.426 | 0.041 |  | -59.727 | 0.000000 | -2.505 | -2.346 | .329 |
|  | Age | 0.002 | 0.001 | 0.019 | 3.008 | 0.002636 | 0.001 | 0.003 |  |
|  | Income | 0.000 | 0.000 | 0.016 | 2.565 | 0.010319 | 0.000 | 0.000 |  |
|  | Wave 1 Envhabit6 | 0.629 | 0.007 | 0.575 | 91.016 | 0.000000 | 0.615 | 0.642 |  |
| 2 | (Constant) | -2.437 | 0.041 |  | -59.49 | 0.000000 | -2.518 | -2.357 | .329 |
|  | Age | 0.002 | 0.001 | 0.022 | 3.295 | 0.000986 | 0.001 | 0.003 |  |
|  | Income | 0.000 | 0.000 | 0.016 | 2.519 | 0.011790 | 0.000 | 0.000 |  |
|  | Wave 1 Envhabit6 | 0.629 | 0.007 | 0.575 | 91.048 | 0.000000 | 0.615 | 0.643 |  |
|  | New Eco-Parent | 0.127 | 0.060 | 0.013 | 2.118 | 0.034224 | 0.009 | 0.245 |  |

| **Model** | **Dependent Variable: Change in envhabit7** | **B** | **SE** | **β** | **t** | **p** | **Lower 95%CI** | **Upper 95%CI** | **R^2^** |
| --- | --- | --- | --- | --- | --- | --- | --- | --- | --- |
| 1 | (Constant) | -2.029 | 0.040 |  | -50.78 | 0.000000 | -2.107 | -1.951 | .257 |
|  | Age | 0.017 | 0.001 | 0.208 | 28.937 | 0.000000 | 0.015 | 0.018 |  |
|  | Income | 0.000 | 0.000 | 0.033 | 4.869 | 0.000001 | 0.000 | 0.000 |  |
|  | Wave 1 Envhabit7 | 0.512 | 0.007 | 0.546 | 77.349 | 0.000000 | 0.499 | 0.524 |  |
| 2 | (Constant) | -2.025 | 0.040 |  | -50.243 | 0.000000 | -2.104 | -1.946 | .257 |
|  | Age | 0.016 | 0.001 | 0.208 | 28.524 | 0.000000 | 0.015 | 0.018 |  |
|  | Income | 0.000 | 0.000 | 0.033 | 4.884 | 0.000001 | 0.000 | 0.000 |  |
|  | Wave 1 Envhabit7 | 0.511 | 0.007 | 0.546 | 77.333 | 0.000000 | 0.499 | 0.524 |  |
|  | New Eco-Parent | -0.048 | 0.060 | -0.005 | -0.788 | 0.430454 | -0.166 | 0.071 |  |

| **Model** | **Dependent Variable: Change in envhabit8** | **B** | **SE** | **β** | **t** | **p** | **Lower 95%CI** | **Upper 95%CI** | **R^2^** |
| --- | --- | --- | --- | --- | --- | --- | --- | --- | --- |
| 1 | (Constant) | -1.702 | 0.033 |  | -52.1 | 0.000000 | -1.766 | -1.638 | .232 |
|  | Age | 0.001 | 0.000 | 0.010 | 1.489 | 0.136443 | 0.000 | 0.002 |  |
|  | Income | 0.000 | 0.000 | -0.019 | -2.636 | 0.008385 | 0.000 | 0.000 |  |
|  | Wave 1 Envhabit8 | 0.405 | 0.006 | 0.483 | 69.788 | 0.000000 | 0.393 | 0.416 |  |
| 2 | (Constant) | -1.696 | 0.033 |  | -51.369 | 0.000000 | -1.760 | -1.631 | .232 |
|  | Age | 0.001 | 0.000 | 0.009 | 1.269 | 0.204346 | 0.000 | 0.002 |  |
|  | Income | 0.000 | 0.000 | -0.018 | -2.606 | 0.009180 | 0.000 | 0.000 |  |
|  | Wave 1 Envhabit8 | 0.405 | 0.006 | 0.483 | 69.755 | 0.000000 | 0.393 | 0.416 |  |
|  | New Eco-Parent | -0.075 | 0.054 | -0.010 | -1.398 | 0.162275 | -0.181 | 0.030 |  |

| **Model** | **Dependent Variable: Change in envhabit9** | **B** | **SE** | **β** | **t** | **p** | **Lower 95%CI** | **Upper 95%CI** | **R^2^** |
| --- | --- | --- | --- | --- | --- | --- | --- | --- | --- |
| 1 | (Constant) | -1.428 | 0.035 |  | -40.83 | 0.000000 | -1.497 | -1.360 | .274 |
|  | Age | -0.006 | 0.001 | -0.074 | -10.969 | 0.000000 | -0.007 | -0.005 |  |
|  | Income | 0.000 | 0.000 | -0.005 | -0.701 | 0.483382 | 0.000 | 0.000 |  |
|  | Wave 1 Envhabit9 | 0.542 | 0.007 | 0.527 | 79.155 | 0.000000 | 0.529 | 0.556 |  |
| 2 | (Constant) | -1.437 | 0.035 |  | -40.657 | 0.000000 | -1.506 | -1.367 | .274 |
|  | Age | -0.006 | 0.001 | -0.072 | -10.605 | 0.000000 | -0.007 | -0.005 |  |
|  | Income | 0.000 | 0.000 | -0.005 | -0.734 | 0.462886 | 0.000 | 0.000 |  |
|  | Wave 1 Envhabit9 | 0.542 | 0.007 | 0.527 | 79.157 | 0.000000 | 0.529 | 0.556 |  |
|  | New Eco-Parent | 0.102 | 0.061 | 0.011 | 1.689 | 0.091241 | -0.016 | 0.221 |  |

| **Model** | **Dependent Variable: Change in envhabit10** | **B** | **SE** | **β** | **t** | **p** | **Lower 95%CI** | **Upper 95%CI** | **R^2^** |
| --- | --- | --- | --- | --- | --- | --- | --- | --- | --- |
| 1 | (Constant) | -2.707 | 0.043 |  | -62.314 | 0.000000 | -2.792 | -2.622 | .394 |
|  | Age | -0.006 | 0.001 | -0.072 | -9.497 | 0.000000 | -0.007 | -0.005 |  |
|  | Income | 0.000 | 0.000 | -0.011 | -1.446 | 0.148203 | 0.000 | 0.000 |  |
|  | Wave 1 Envhabit10 | 0.707 | 0.008 | 0.638 | 85.594 | 0.000000 | 0.691 | 0.723 |  |
| 2 | (Constant) | -2.700 | 0.044 |  | -61.633 | 0.000000 | -2.786 | -2.615 | .394 |
|  | Age | -0.006 | 0.001 | -0.073 | -9.565 | 0.000000 | -0.007 | -0.005 |  |
|  | Income | 0.000 | 0.000 | -0.011 | -1.427 | 0.153578 | 0.000 | 0.000 |  |
|  | Wave 1 Envhabit10 | 0.707 | 0.008 | 0.638 | 85.599 | 0.000000 | 0.691 | 0.723 |  |
|  | New Eco-Parent | -0.075 | 0.064 | -0.009 | -1.172 | 0.241228 | -0.201 | 0.051 |  |

| **Model** | **Dependent Variable: Change in envhabit11** | **B** | **SE** | **β** | **t** | **p** | **Lower 95%CI** | **Upper 95%CI** | **R^2^** |
| --- | --- | --- | --- | --- | --- | --- | --- | --- | --- |
| 1 | (Constant) | -3.390 | 0.051 |  | -66.264 | 0.000000 | -3.490 | -3.289 | .493 |
|  | Age | -0.003 | 0.001 | -0.036 | -4.491 | 0.000007 | -0.004 | -0.002 |  |
|  | Income | 0.000 | 0.000 | -0.006 | -0.732 | 0.464157 | 0.000 | 0.000 |  |
|  | Wave 1 Envhabit11 | 0.766 | 0.009 | 0.703 | 88.362 | 0.000000 | 0.749 | 0.783 |  |
| 2 | (Constant) | -3.401 | 0.052 |  | -66.039 | 0.000000 | -3.502 | -3.300 | .493 |
|  | Age | -0.003 | 0.001 | -0.034 | -4.166 | 0.000031 | -0.004 | -0.002 |  |
|  | Income | 0.000 | 0.000 | -0.007 | -0.81 | 0.418171 | 0.000 | 0.000 |  |
|  | Wave 1 Envhabit11 | 0.767 | 0.009 | 0.703 | 88.39 | 0.000000 | 0.750 | 0.784 |  |
|  | New Eco-Parent | 0.136 | 0.070 | 0.016 | 1.941 | 0.052287 | -0.001 | 0.274 |  |

## New Mother Status

| **Model** | **Dependent Variable:Change in ftst** | **B** | **SE** | **β** | **t** | **p** | **Lower 95%CI** | **Upper 95%CI** | **R^2^** |
| --- | --- | --- | --- | --- | --- | --- | --- | --- | --- |
| 1 | (Constant) | 1.019 | 0.017 |  | 60.706 | 0.000000 | 0.986 | 1.052 | .332 |
|  | Age | -0.003 | 0.000 | -0.083 | -13.184 | 0.000000 | -0.003 | -0.002 |  |
|  | Income | 0.000 | 0.000 | 0.012 | 2.003 | 0.045163 | 0.000 | 0.000 |  |
|  | Wave 1 Ftst | -0.650 | 0.007 | -0.584 | -94.55 | 0.000000 | -0.664 | -0.637 |  |
| 2 | (Constant) | 1.020 | 0.017 |  | 60.373 | 0.000000 | 0.987 | 1.053 | .332 |
|  | Age | -0.003 | 0.000 | -0.083 | -13.133 | 0.000000 | -0.003 | -0.003 |  |
|  | Income | 0.000 | 0.000 | 0.012 | 2.005 | 0.044995 | 0.000 | 0.000 |  |
|  | Wave 1 Ftst | -0.650 | 0.007 | -0.584 | -94.547 | 0.000000 | -0.664 | -0.637 |  |
|  | New Mother | -0.014 | 0.028 | -0.003 | -0.482 | 0.629971 | -0.069 | 0.042 |  |

| **Model** | **Dependent Variable: Change in crlf** | **B** | **SE** | **β** | **t** | **p** | **Lower 95%CI** | **Upper 95%CI** | **R^2^** |
| --- | --- | --- | --- | --- | --- | --- | --- | --- | --- |
| 1 | (Constant) | 1.454 | 0.025 |  | 58.844 | 0.000000 | 1.406 | 1.503 | .302 |
|  | Age | 0.005 | 0.000 | 0.103 | 15.723 | 0.000000 | 0.005 | 0.006 |  |
|  | Income | 0.000 | 0.000 | 0.002 | 0.369 | 0.711800 | 0.000 | 0.000 |  |
|  | Wave 1 crlf | -0.623 | 0.007 | -0.562 | -88.074 | 0.000000 | -0.637 | -0.609 |  |
| 2 | (Constant) | 1.461 | 0.025 |  | 58.717 | 0.000000 | 1.412 | 1.510 | .302 |
|  | Age | 0.005 | 0.000 | 0.101 | 15.289 | 0.000000 | 0.005 | 0.006 |  |
|  | Income | 0.000 | 0.000 | 0.002 | 0.376 | 0.706923 | 0.000 | 0.000 |  |
|  | Wave 1 crlf | -0.623 | 0.007 | -0.562 | -88.082 | 0.000000 | -0.637 | -0.609 |  |
|  | New Mother | -0.099 | 0.043 | -0.015 | -2.313 | 0.020757 | -0.183 | -0.015 |  |

| **Model** | **Dependent Variable: Change in grn** | **B** | **SE** | **β** | **t** | **p** | **Lower 95%CI** | **Upper 95%CI** | **R^2^** |
| --- | --- | --- | --- | --- | --- | --- | --- | --- | --- |
| 1 | (Constant) | 1.671 | 0.023 |  | 71.308 | 0.000000 | 1.625 | 1.717 | .334 |
|  | Age | -0.002 | 0.000 | -0.044 | -6.995 | 0.000000 | -0.003 | -0.001 |  |
|  | Income | 0.000 | 0.000 | 0.039 | 6.2 | 0.000000 | 0.000 | 0.000 |  |
|  | Wave 1 grn | -0.638 | 0.007 | -0.586 | -93.864 | 0.000000 | -0.652 | -0.625 |  |
| 2 | (Constant) | 1.671 | 0.024 |  | 70.909 | 0.000000 | 1.625 | 1.717 | .334 |
|  | Age | -0.002 | 0.000 | -0.044 | -6.918 | 0.000000 | -0.003 | -0.001 |  |
|  | Income | 0.000 | 0.000 | 0.039 | 6.199 | 0.000000 | 0.000 | 0.000 |  |
|  | Wave 1 grn | -0.638 | 0.007 | -0.586 | -93.861 | 0.000000 | -0.652 | -0.625 |  |
|  | New Mother | 0.004 | 0.035 | 0.001 | 0.118 | 0.906426 | -0.065 | 0.074 |  |

| **Model** | **Dependent Variable: Change in envhabit1** | **B** | **SE** | **β** | **t** | **p** | **Lower 95%CI** | **Upper 95%CI** | **R^2^** |
| --- | --- | --- | --- | --- | --- | --- | --- | --- | --- |
| 1 | (Constant) | 1.276 | 0.048 |  | 26.806 | 0.000000 | 1.183 | 1.370 | .236 |
|  | Age | 0.008 | 0.001 | 0.077 | 11.373 | 0.000000 | 0.007 | 0.009 |  |
|  | Income | 0.000 | 0.000 | -0.026 | -3.856 | 0.000116 | 0.000 | 0.000 |  |
|  | Wave 1 Envhabit1 | -0.539 | 0.007 | -0.488 | -73.904 | 0.000000 | -0.553 | -0.524 |  |
| 2 | (Constant) | 1.277 | 0.048 |  | 26.559 | 0.000000 | 1.183 | 1.372 | .236 |
|  | Age | 0.008 | 0.001 | 0.077 | 11.258 | 0.000000 | 0.007 | 0.009 |  |
|  | Income | 0.000 | 0.000 | -0.026 | -3.855 | 0.000116 | 0.000 | 0.000 |  |
|  | Wave 1 Envhabit1 | -0.539 | 0.007 | -0.488 | -73.879 | 0.000000 | -0.553 | -0.524 |  |
|  | New Mother | -0.011 | 0.088 | -0.001 | -0.122 | 0.902789 | -0.184 | 0.162 |  |

| **Model** | **Dependent Variable: Change in envhabit2** | **B** | **SE** | **β** | **t** | **p** | **Lower 95%CI** | **Upper 95%CI** | **R^2^** |
| --- | --- | --- | --- | --- | --- | --- | --- | --- | --- |
| 1 | (Constant) | -1.237 | 0.025 |  | -50.012 | 0.000000 | -1.285 | -1.188 | .398 |
|  | Age | 0.003 | 0.000 | 0.053 | 9.001 | 0.000000 | 0.003 | 0.004 |  |
|  | Income | 0.000 | 0.000 | -0.034 | -5.758 | 0.000000 | 0.000 | 0.000 |  |
|  | Wave 1 Envhabit2 | 0.730 | 0.007 | 0.638 | 109.283 | 0.000000 | 0.717 | 0.743 |  |
| 2 | (Constant) | -1.229 | 0.025 |  | -49.243 | 0.000000 | -1.277 | -1.180 | .398 |
|  | Age | 0.003 | 0.000 | 0.051 | 8.584 | 0.000000 | 0.002 | 0.004 |  |
|  | Income | 0.000 | 0.000 | -0.034 | -5.75 | 0.000000 | 0.000 | 0.000 |  |
|  | Wave 1 Envhabit2 | 0.730 | 0.007 | 0.637 | 109.264 | 0.000000 | 0.717 | 0.743 |  |
|  | New Mother | -0.118 | 0.047 | -0.015 | -2.522 | 0.011669 | -0.209 | -0.026 |  |

| **Model** | **Dependent Variable: Change in envhabit3** | **B** | **SE** | **β** | **t** | **p** | **Lower 95%CI** | **Upper 95%CI** | **R^2^** |
| --- | --- | --- | --- | --- | --- | --- | --- | --- | --- |
| 1 | (Constant) | 1.335 | 0.039 |  | 34.027 | 0.000000 | 1.258 | 1.411 | .240 |
|  | Age | 0.005 | 0.001 | 0.052 | 7.851 | 0.000000 | 0.004 | 0.006 |  |
|  | Income | 0.000 | 0.000 | 0.023 | 3.449 | 0.000564 | 0.000 | 0.000 |  |
|  | Wave 1 Envhabit3 | -0.459 | 0.006 | -0.489 | -75.102 | 0.000000 | -0.471 | -0.447 |  |
| 2 | (Constant) | 1.344 | 0.040 |  | 33.956 | 0.000000 | 1.266 | 1.421 | .240 |
|  | Age | 0.005 | 0.001 | 0.051 | 7.551 | 0.000000 | 0.003 | 0.006 |  |
|  | Income | 0.000 | 0.000 | 0.023 | 3.454 | 0.000553 | 0.000 | 0.000 |  |
|  | Wave 1 Envhabit3 | -0.459 | 0.006 | -0.489 | -75.116 | 0.000000 | -0.471 | -0.447 |  |
|  | New Mother | -0.132 | 0.076 | -0.011 | -1.741 | 0.081694 | -0.280 | 0.017 |  |

| **Model** | **Dependent Variable: Change in envhabit4** | **B** | **SE** | **β** | **t** | **p** | **Lower 95%CI** | **Upper 95%CI** | **R^2^** |
| --- | --- | --- | --- | --- | --- | --- | --- | --- | --- |
| 1 | (Constant) | -1.549 | 0.034 |  | -45.55 | 0.000000 | -1.616 | -1.483 | .334 |
|  | Age | -0.001 | 0.001 | -0.014 | -2.226 | 0.026021 | -0.002 | 0.000 |  |
|  | Income | 0.000 | 0.000 | -0.003 | -0.553 | 0.580469 | 0.000 | 0.000 |  |
|  | Wave 1 Envhabit4 | 0.644 | 0.007 | 0.577 | 94.847 | 0.000000 | 0.631 | 0.657 |  |
| 2 | (Constant) | -1.546 | 0.034 |  | -45.119 | 0.000000 | -1.613 | -1.479 | .334 |
|  | Age | -0.001 | 0.001 | -0.014 | -2.308 | 0.020991 | -0.002 | 0.000 |  |
|  | Income | 0.000 | 0.000 | -0.003 | -0.551 | 0.581767 | 0.000 | 0.000 |  |
|  | Wave 1 Envhabit4 | 0.644 | 0.007 | 0.577 | 94.849 | 0.000000 | 0.631 | 0.657 |  |
|  | New Mother | -0.049 | 0.064 | -0.005 | -0.767 | 0.443145 | -0.175 | 0.077 |  |

| **Model** | **Dependent Variable: Change in envhabit5** | **B** | **SE** | **β** | **t** | **p** | **Lower 95%CI** | **Upper 95%CI** | **R^2^** |
| --- | --- | --- | --- | --- | --- | --- | --- | --- | --- |
| 1 | (Constant) | -2.766 | 0.037 |  | -75.333 | 0.000000 | -2.838 | -2.694 | .341 |
|  | Age | 0.000 | 0.000 | 0.000 | 0.002 | 0.998725 | -0.001 | 0.001 |  |
|  | Income | 0.000 | 0.000 | 0.015 | 2.476 | 0.013287 | 0.000 | 0.000 |  |
|  | Wave 1 Envhabit5 | 0.625 | 0.007 | 0.584 | 95.184 | 0.000000 | 0.612 | 0.638 |  |
| 2 | (Constant) | -2.770 | 0.037 |  | -75.136 | 0.000000 | -2.842 | -2.697 | .341 |
|  | Age | 0.000 | 0.000 | 0.001 | 0.132 | 0.894591 | -0.001 | 0.001 |  |
|  | Income | 0.000 | 0.000 | 0.015 | 2.471 | 0.013485 | 0.000 | 0.000 |  |
|  | Wave 1 Envhabit5 | 0.625 | 0.007 | 0.584 | 95.184 | 0.000000 | 0.612 | 0.638 |  |
|  | New Mother | 0.047 | 0.048 | 0.006 | 0.997 | 0.318543 | -0.046 | 0.141 |  |

| **Model** | **Dependent Variable: Change in envhabit6** | **B** | **SE** | **β** | **t** | **p** | **Lower 95%CI** | **Upper 95%CI** | **R^2^** |
| --- | --- | --- | --- | --- | --- | --- | --- | --- | --- |
| 1 | (Constant) | -2.420 | 0.041 |  | -59.735 | 0.000000 | -2.499 | -2.340 | .329 |
|  | Age | 0.002 | 0.001 | 0.019 | 2.909 | 0.003626 | 0.001 | 0.003 |  |
|  | Income | 0.000 | 0.000 | 0.016 | 2.503 | 0.012309 | 0.000 | 0.000 |  |
|  | Wave 1 Envhabit6 | 0.628 | 0.007 | 0.575 | 91.058 | 0.000000 | 0.615 | 0.642 |  |
| 2 | (Constant) | -2.420 | 0.041 |  | -59.374 | 0.000000 | -2.500 | -2.340 | .329 |
|  | Age | 0.002 | 0.001 | 0.019 | 2.9 | 0.003734 | 0.001 | 0.003 |  |
|  | Income | 0.000 | 0.000 | 0.016 | 2.503 | 0.012340 | 0.000 | 0.000 |  |
|  | Wave 1 Envhabit6 | 0.628 | 0.007 | 0.575 | 91.055 | 0.000000 | 0.615 | 0.642 |  |
|  | New Mother | 0.008 | 0.067 | 0.001 | 0.126 | 0.900108 | -0.123 | 0.139 |  |

| **Model** | **Dependent Variable: Change in envhabit7** | **B** | **SE** | **β** | **t** | **p** | **Lower 95%CI** | **Upper 95%CI** | **R^2^** |
| --- | --- | --- | --- | --- | --- | --- | --- | --- | --- |
| 1 | (Constant) | -2.028 | 0.040 |  | -50.803 | 0.000000 | -2.106 | -1.949 | .257 |
|  | Age | 0.016 | 0.001 | 0.208 | 28.928 | 0.000000 | 0.015 | 0.018 |  |
|  | Income | 0.000 | 0.000 | 0.032 | 4.765 | 0.000002 | 0.000 | 0.000 |  |
|  | Wave 1 Envhabit7 | 0.512 | 0.007 | 0.547 | 77.508 | 0.000000 | 0.499 | 0.525 |  |
| 2 | (Constant) | -2.022 | 0.040 |  | -50.438 | 0.000000 | -2.100 | -1.943 | .258 |
|  | Age | 0.016 | 0.001 | 0.207 | 28.577 | 0.000000 | 0.015 | 0.018 |  |
|  | Income | 0.000 | 0.000 | 0.032 | 4.778 | 0.000002 | 0.000 | 0.000 |  |
|  | Wave 1 Envhabit7 | 0.512 | 0.007 | 0.547 | 77.525 | 0.000000 | 0.499 | 0.525 |  |
|  | New Mother | -0.105 | 0.068 | -0.010 | -1.559 | 0.118985 | -0.238 | 0.027 |  |

| **Model** | **Dependent Variable: Change in envhabit8** | **B** | **SE** | **β** | **t** | **p** | **Lower 95%CI** | **Upper 95%CI** | **R^2^** |
| --- | --- | --- | --- | --- | --- | --- | --- | --- | --- |
| 1 | (Constant) | -1.705 | 0.033 |  | -52.357 | 0.000000 | -1.769 | -1.641 | .232 |
|  | Age | 0.001 | 0.000 | 0.011 | 1.531 | 0.125693 | 0.000 | 0.002 |  |
|  | Income | 0.000 | 0.000 | -0.018 | -2.589 | 0.009645 | 0.000 | 0.000 |  |
|  | Wave 1 Envhabit8 | 0.405 | 0.006 | 0.483 | 69.878 | 0.000000 | 0.394 | 0.416 |  |
| 2 | (Constant) | -1.706 | 0.033 |  | -51.972 | 0.000000 | -1.770 | -1.641 | .232 |
|  | Age | 0.001 | 0.000 | 0.011 | 1.545 | 0.122460 | 0.000 | 0.002 |  |
|  | Income | 0.000 | 0.000 | -0.018 | -2.589 | 0.009631 | 0.000 | 0.000 |  |
|  | Wave 1 Envhabit8 | 0.405 | 0.006 | 0.483 | 69.875 | 0.000000 | 0.394 | 0.416 |  |
|  | New Mother | 0.012 | 0.059 | 0.001 | 0.202 | 0.839831 | -0.104 | 0.128 |  |

| **Model** | **Dependent Variable: Change in envhabit9** | **B** | **SE** | **β** | **t** | **p** | **Lower 95%CI** | **Upper 95%CI** | **R^2^** |
| --- | --- | --- | --- | --- | --- | --- | --- | --- | --- |
| 1 | (Constant) | -1.428 | 0.035 |  | -40.919 | 0.000000 | -1.497 | -1.360 | .275 |
|  | Age | -0.006 | 0.001 | -0.075 | -11.093 | 0.000000 | -0.007 | -0.005 |  |
|  | Income | 0.000 | 0.000 | -0.005 | -0.71 | 0.477752 | 0.000 | 0.000 |  |
|  | Wave 1 Envhabit9 | 0.543 | 0.007 | 0.528 | 79.387 | 0.000000 | 0.530 | 0.557 |  |
| 2 | (Constant) | -1.431 | 0.035 |  | -40.631 | 0.000000 | -1.500 | -1.362 | .275 |
|  | Age | -0.006 | 0.001 | -0.074 | -10.916 | 0.000000 | -0.007 | -0.005 |  |
|  | Income | 0.000 | 0.000 | -0.005 | -0.712 | 0.476471 | 0.000 | 0.000 |  |
|  | Wave 1 Envhabit9 | 0.543 | 0.007 | 0.528 | 79.384 | 0.000000 | 0.530 | 0.557 |  |
|  | New Mother | 0.039 | 0.066 | 0.004 | 0.59 | 0.555398 | -0.091 | 0.169 |  |

| **Model** | **Dependent Variable: Change in envhabit10** | **B** | **SE** | **β** | **t** | **p** | **Lower 95%CI** | **Upper 95%CI** | **R^2^** |
| --- | --- | --- | --- | --- | --- | --- | --- | --- | --- |
| 1 | (Constant) | -2.709 | 0.043 |  | -62.446 | 0.000000 | -2.794 | -2.624 | .394 |
|  | Age | -0.006 | 0.001 | -0.072 | -9.474 | 0.000000 | -0.007 | -0.005 |  |
|  | Income | 0.000 | 0.000 | -0.010 | -1.412 | 0.158073 | 0.000 | 0.000 |  |
|  | Wave 1 Envhabit10 | 0.707 | 0.008 | 0.639 | 85.695 | 0.000000 | 0.691 | 0.723 |  |
| 2 | (Constant) | -2.709 | 0.044 |  | -61.965 | 0.000000 | -2.794 | -2.623 | .394 |
|  | Age | -0.006 | 0.001 | -0.072 | -9.405 | 0.000000 | -0.007 | -0.005 |  |
|  | Income | 0.000 | 0.000 | -0.010 | -1.412 | 0.158107 | 0.000 | 0.000 |  |
|  | Wave 1 Envhabit10 | 0.707 | 0.008 | 0.639 | 85.692 | 0.000000 | 0.691 | 0.723 |  |
|  | New Mother | -0.009 | 0.071 | -0.001 | -0.124 | 0.901583 | -0.147 | 0.130 |  |

| **Model** | **Dependent Variable: Change in envhabit11** | **B** | **SE** | **β** | **t** | **p** | **Lower 95%CI** | **Upper 95%CI** | **R^2^** |
| --- | --- | --- | --- | --- | --- | --- | --- | --- | --- |
| 1 | (Constant) | -3.390 | 0.051 |  | -66.329 | 0.000000 | -3.490 | -3.289 | .493 |
|  | Age | -0.003 | 0.001 | -0.036 | -4.509 | 0.000007 | -0.004 | -0.002 |  |
|  | Income | 0.000 | 0.000 | -0.006 | -0.725 | 0.468407 | 0.000 | 0.000 |  |
|  | Wave 1 Envhabit11 | 0.766 | 0.009 | 0.703 | 88.428 | 0.000000 | 0.749 | 0.783 |  |
| 2 | (Constant) | -3.386 | 0.051 |  | -65.777 | 0.000000 | -3.487 | -3.285 | .493 |
|  | Age | -0.003 | 0.001 | -0.037 | -4.548 | 0.000005 | -0.004 | -0.002 |  |
|  | Income | 0.000 | 0.000 | -0.006 | -0.715 | 0.474420 | 0.000 | 0.000 |  |
|  | Wave 1 Envhabit11 | 0.766 | 0.009 | 0.703 | 88.404 | 0.000000 | 0.749 | 0.783 |  |
|  | New Mother | -0.046 | 0.077 | -0.005 | -0.599 | 0.549368 | -0.197 | 0.105 |  |
